# Serum Escape Landscape of SARS-CoV-2 Omicron JN.1 and XEC RBD Under COVID-19 Vaccine Breakthrough Immunity in China

**DOI:** 10.3390/microorganisms14091872

**Published:** 2026-08-23

**Authors:** Chengwei Shao, Jianguang Fu, Fei Deng, Huiyan Yu, Huan Fan, Yanjun Chen, Ke Xu, Mingwei Wei, Siyue Jia, Xiaoyan Jia, Liguo Zhu, Jingxin Li

**Affiliations:** 1 School of Public Health, Southeast University, Nanjing 210009, China; 2 Jiangsu Provincial Medical Innovation Center, National Health Commission Key Laboratory of Enteric Pathogenic Microbiology, Jiangsu Provincial Center for Disease Control and Prevention (Jiangsu Provincial Academy of Preventive Medicine), Nanjing 210009, China; 3 School of Public Health, National Vaccine Innovation Platform, Nanjing Medical University, Nanjing 211166, China; 4 Institute of Pediatrics, Guangzhou Women and Children’s Medical Center, Guangzhou Medical University, Guangzhou 511300, China; 5 Taizhou Institute of Pharmaceutical Industry, Nanjing Medical University, Taizhou 225300, China

**Keywords:** SARS-CoV-2, receptor-binding domain, deep mutational scanning

## Abstract

Population immune pressure from vaccination and prior infection continues to drive the evolution of SARS-CoV-2. Systematic characterization of RBD mutations under complex immune backgrounds is essential for understanding viral adaptation and evolutionary trajectories. Here, we applied a deep mutational scanning (DMS) to comprehensively map the neutralization escape landscape of the Omicron variant JN.1 and its descendant lineage XEC, under immune pressure from individuals who experienced Omicron breakthrough infections following three doses of inactivated vaccines. A neutralization escape map for the single amino acid substitutions in the RBD of JN.1 or XEC was generated, and the escape efficiency of each mutation was determined. The results show that RBD escape mutations are hierarchically organized: low-intensity signals are widespread, whereas high-intensity escape is confined to a few key sites. These escape mutations are not confined solely to the receptor-binding motif (RBM) but are broadly distributed across the entire RBD. Many escape sites could accommodate multiple amino acid substitutions. Integration of DMS data with genomic surveillance of circulating variants from 2024 to 2025 revealed significant overlap between experimentally identified escape sites and mutations observed in natural isolates. This overlap increased substantially in 2025, with site concordance rising from 27.17% and 26.81% to 45.09% and 47.10% for JN.1 and XEC, respectively. The natural prevalence of these escape mutations is further shaped by factors such as receptor-binding affinity, protein stability, and epistatic interactions. Overall, our findings suggest that SARS-CoV-2 antigenic evolution follows the pattern of multiple pathways within a constrained space, providing new insights into the adaptive mechanisms of Omicron-derived variants under hybrid immune pressure.

## 1. Introduction

Since the emergence of severe acute respiratory syndrome coronavirus 2 (SARS-CoV-2), repeated vaccination and infection have progressively shaped a complex population immune landscape, driving the virus to accumulate mutations that balance infectivity and immune escape [1,2,3]. The Omicron variant and its descendants, particularly within the receptor-binding domain (RBD), have acquired numerous amino acid substitutions that alter angiotensin-converting enzyme 2 (ACE2) binding and evade neutralizing antibodies [4,5,6,7,8]. Therefore, systematically characterizing the functional effects of RBD mutations under this immune background is essential for understanding current and future evolutionary trajectories of the virus.

Traditional neutralization assays using pseudovirus or live virus are limited in throughput and cannot comprehensively assess the vast mutational space [9,10,11]. Deep mutational scanning (DMS) employs saturated mutagenesis libraries coupled with selective screening and high-throughput sequencing, enabling the systematic and high-throughput analysis of the functional impact of nearly all possible amino acid substitutions within a protein [12]. In recent years, DMS has been widely applied to studies of SARS-CoV-2 mutations, systematically revealing their effects on ACE2 binding, protein expression and stability, escape from monoclonal antibodies, and antibody-binding or neutralization escape from polyclonal sera [7,13,14,15,16,17,18,19]. DMS studies based on full-length spike and pseudovirus systems have further enhanced the functional assessment of mutation-mediated effects on viral entry and antibody escape [7,19].

In our previous study, we employed a two-step, non-replicating pseudovirus-based DMS platform and demonstrated its ability to systematically characterize the effects of RBD mutations in the JN.1 and XEC spike backgrounds on spike-mediated viral entry and monoclonal antibody escape. That study focused primarily on the application and validation of the experimental platform and the generation of comprehensive functional maps of mutations [20]. Building on this methodological foundation, the present study shifts its focus to applications within the context of real-world population immunity. Population immune backgrounds have become increasingly complex and heterogeneous across regions because of differences in vaccination strategies and repeated natural and breakthrough infections [2,4]. Consequently, it remains necessary to clarify how different Omicron genetic backgrounds and distinct immune exposure histories modulate the escape effects of individual mutations. Moreover, the relationship between population immunity in specific settings and the accumulation and evolution of genomic mutations in circulating viruses remains insufficiently understood.

Building on this foundation, this study systematically evaluated serum neutralization escape of the Omicron variant JN.1 and its descendant lineage XEC from population immune background shaped by breakthrough Omicron infections following three doses of inactivated COVID-19 vaccines. Combined with real-world genomic surveillance data of circulating SARS-CoV-2 variants during 2024–2025, we further compared escape mutations identified via DMS with those detected in epidemiological surveillance.

## 2. Materials and Methods

### 2.1. Serum and Ethics Statement

Archived serum samples used in this study were obtained from participants enrolled in two randomized clinical trials: “Study on Sequential Immunization of Inactivated SARS-CoV-2 Vaccine and Recombinant SARS-CoV-2 Vaccine (Ad5 Vector)” and “Study on Sequential Immunization of Inactivated COVID-19 Vaccine and Recombinant COVID-19 Vaccine (Ad5 Vector) in Elderly Adults,” registered at ClinicalTrials.gov (NCT04892459 and NCT04952727). The participants included in the present study had received three doses of inactivated COVID-19 vaccine and subsequently experienced SARS-CoV-2 Omicron breakthrough infection. A total of 40 serum samples were included, and every four individual sera were pooled at equal volumes to generate one pooled serum sample, resulting in 10 pooled serum samples for neutralization and DMS escape assays. Serum samples were collected approximately seven months after breakthrough infection, between 16 July and 24 July 2023. All samples and associated data were de-identified before analysis, and the authors did not have access to information that could directly identify individual participants during or after the present analysis. The project was approved by the Ethics Committee of Jiangsu Provincial Center for Disease Prevention and Control (Approval No. JSJK2023-B003-02). Written informed consent was obtained from all participants at enrollment and before sample collection. All procedures were conducted in accordance with the Declaration of Helsinki and relevant ethical guidelines and regulations.

### 2.2. Cell Lines and Culture

We generated a HEK-293T-derived stable cell line expressing the reverse tetracycline-controlled transactivator (rtTA) (HEK-293T-rtTA) as described by Dadonaite et al., to support the inducible expression system used in DMS experiments [19]. This cell line was used for stable maintenance of the mutant libraries and for induction of gene expression driven by the TRE3G promoter. All cell lines used in this study (HEK-293T, HEK-293T-ACE2, and HEK-293T-rtTA) were maintained in complete DMEM medium consisting of high-glucose DMEM (containing 4.5 g/L D-glucose, 2 mM L-glutamine, and 110 mg/L sodium pyruvate) (Gibco, Waltham, MA, USA, C11995500BT), supplemented with 10% fetal bovine serum (Gibco, Waltham, MA, USA, A5256701) and 100 U/mL penicillin-streptomycin (Gibco, Waltham, MA, USA, 15070063). All cells were maintained in a 37 °C, 5% CO_2_ incubator and passaged routinely every 2–3 days.

### 2.3. Spike Gene Modification and Lentiviral Vector Construction

The coding sequence of the spike protein from the JN.1 variant used in this study was synthesized by GenScript (Nanjing, China) and subjected to quality control and sequence verification. The spike gene of the XEC variant was generated based on the JN.1 spike sequence by introducing substitution mutations at two key amino acid positions, T22N and F59S. These substitutions were introduced using overlapping extension PCR primers and constructed through a site-directed mutagenesis strategy.

These spike genes were cloned into the replication-deficient lentiviral backbone previously described by Dadonaite et al. [19]. Briefly, the vector backbone was amplified by PCR, and the template plasmid was removed before homologous recombination assembly with the insert fragment. The recombinant products were transformed into Stbl3 competent cells (ALpaLifeBio, Shenzhen, China, KTSM110L), and positive clones were selected by 100 μg/mL ampicillin (Beyotime, Shanghai, China, ST008). The accuracy of the constructs and the integrity of the plasmids were confirmed by NGS. Assembly of infectious lentiviral particles depends on co-transfection with expression plasmid and helper plasmids (including Gag/Pol, Tat1b, and Rev1b), which provide the necessary structural proteins and replication-associated factors to enable viral particle production in packaging cells. The spike coding sequence was placed downstream of the inducible TRE3G promoter, with its expression regulated by rtTA. Addition of doxycycline (MCE, Monmouth Junction, New Jersey, USA, HY-N0565) to HEK-293T-rtTA cell cultures activates TRE3G promoter activity, thereby inducing spike protein transcription. The ZsGreen fluorescent reporter and the puromycin resistance gene were driven by the CMV promoter and were used to assess viral infection efficiency and to select stably transduced cell populations, respectively.

### 2.4. Design of DMS Library

All mutagenic oligonucleotides were synthesized by NEOPRIMARIES (BGI, Shenzhen, China). The Wuhan-Hu-1 sequence was used as the reference for amino acid numbering. The RBD of the JN.1 spike protein was defined as residues R319 to F541. As JN.1 and XEC share an identical amino acid sequence within the RBD, a single mutant library was constructed for use in both variants. We divided the 666 bp RBD coding region of the JN.1 spike gene into four fragments (3 × 168 bp and 1 × 162 bp, corresponding to 56 and 54 amino acid sites, respectively) to accommodate sequencing read-length limitations. A 25 bp homologous sequence was introduced at both ends of each fragment to facilitate mutant library construction. The single-codon substitution strategy was employed, whereby each codon was mutated to the other 63 possible codons (including synonymous and stop codons), while retaining the wild-type sequence as a control.

### 2.5. Construction of the DMS Plasmid Library

We constructed the DMS plasmid library with a minimum of 10-fold theoretical coverage at the cloning stage to achieve the desired mutation complexity. Mutant fragments were seamlessly cloned into the spike coding region within the lentiviral backbone. The recombinant products were transformed into Stbl3 competent cells. A part of the transformation mixture was plated onto LB agar plates containing 100 μg/mL ampicillin and incubated overnight at 30 °C. Colony counts were used to estimate library complexity. The remaining bacterial culture was expanded for plasmid extraction. We performed Tn5 transposase-mediated sequencing to verify vector backbone integrity and confirm library suitability for subsequent cell-based experiments. Amplicon sequencing was further conducted to assess mutational coverage and distribution uniformity across the library.

### 2.6. Generation of the DMS Cell Library

The construction process of the cell storage spike protein DMS library followed previously described protocols [19]. Briefly, the DMS plasmid library was co-transfected into HEK-293T cells together with the helper plasmids (HDM-Hgpm2, Addgene #204152; HDM-Tat1b, Addgene #204154; pRC-CMV-Rev1b, Addgene #204153) and VSV-G expression plasmid (Addgene #204156), generating the VSV-G–pseudotyped lentiviral library carrying mutant spike sequences. HEK-293T-rtTA cells were then infected with VSV-G pseudotyped lentiviral library at low multiplicity of infection (MOI), thus ensuring that each cell integrates at most one mutant vector. Following infection, puromycin selection was applied to enrich for cells with stable vector integration until all surviving cells expressed the ZsGreen fluorescent reporter. The part of cell population was collected for amplicon sequencing to further evaluate mutational coverage and distribution uniformity within the library.

### 2.7. Pseudovirus Neutralization Assay

All serum samples were heat-treated at 56 °C for 1 h prior to the assay to inactivate complement activity. Based on pre-determined viral titers, the amount of pseudovirus used in each batch of experiments was standardized to ensure comparability between experiments. Sera were subjected to 4-fold serial dilutions in 96-well plates, starting at a dilution of 1:20, with two technical replicates for each dilution. The diluted pseudovirus was then added to each well at a 1:1 volume ratio and incubated with sera at 37 °C for 1 h. Following incubation, 2 × 10^4^ HEK-293T-ACE2 cells were added to each well, and the plates were cultured at 37 °C for approximately 48 h. Infection levels were quantified by measuring luciferase activity using a luciferase assay kit (Beyotime, Shanghai, China, RG042S) according to the manufacturer’s instructions. Data were read and quantitatively analyzed using SoftMax Pro v7.0.2 (Molecular Devices) software. Neutralization curves were fitted using a five-parameter nonlinear dose–response model in GraphPad Prism v9.2 and all neutralization-related statistical computations were performed in GraphPad Prism v9.2, include IC_50_ and IC_80_ values.

### 2.8. Serum Escape Assay

Two systems were set up in the serum neutralization escape assay: spike-pseudotyped lentivirus and VSV-G-pseudotyped lentivirus. For each system, a serum-treated group and a no-serum control group were included. For each pooled serum sample, the DMS escape assay included spike-pseudotyped lentivirus with serum, spike-pseudotyped lentivirus without serum, VSV-G-pseudotyped lentivirus with serum, and VSV-G-pseudotyped lentivirus without serum. Each condition was performed with two independent biological replicates. The serum dilution used in the escape assay was determined based on the IC_80_ obtained from the standard pseudovirus neutralization assay. This selection strength effectively distinguished escape mutations while avoiding excessive stringency that could lead to large-scale loss of variants, thereby maintaining the resolvability of library variant information. Diluted serum was mixed with pseudovirus suspension at an equal volume ratio and incubated at 37 °C with 5% CO_2_ for 1 h. Subsequently, 1 × 10^6^ HEK-293T-ACE2 cells (for spike-pseudotyped lentivirus) or HEK-293T cells (for VSV-G–pseudotyped lentivirus) were seeded into each well of a 6-well plate and infected with the virus-serum mixture. At 24 h post-infection, cells were harvested for genomic DNA extraction. The full-length spike coding region was amplified by PCR, and Illumina amplicon libraries were prepared for subsequent sequencing analysis. Control groups were infected under serum-free conditions following the same procedures and timeline as the serum-treated groups.

### 2.9. NGS Sample Processing

Two complementary sequencing strategies were employed in this study: Tn5-based random fragmentation sequencing and targeted amplicon sequencing. Tn5 sequencing was performed to evaluate the integrity of the full-length lentiviral plasmid backbone, ensuring that no large deletions or unintended mutations were introduced during the cloning process. Briefly, DNA was randomly fragmented and barcoded using Tn5 transposase (abclonal, Wuhan, China, RK20547), followed by PCR amplification to construct sequencing libraries. The amplified products were purified and quantified using magnetic beads (abclonal, Wuhan, China, RK20257) for sequencing on the Illumina platform.

We constructed targeted amplicon sequencing libraries for the RBD region of interest to further assess mutational coverage and distribution uniformity within the mutant library. A two-step PCR protocol was used to generate the amplicon libraries with KAPA HiFi HotStart DNA Polymerase (KAPA, Basel, Switzerland, KK2502). In the first round, the target fragments were amplified and partial sequencing adaptor sequences were introduced. In the second round, full dual indices (p5 and p7) were added to enable sample multiplexing. The final PCR products were purified and subjected to high-throughput sequencing.

### 2.10. NGS Data Processing

#### 2.10.1. Overview

To systematically analyze the DMS data, a modular data-processing pipeline was established to enable unified analysis of different types of sequencing datasets. The workflow comprised two major components: one for evaluating plasmid backbone integrity using Tn5-based random fragmentation sequencing data, and the other for quantifying variant frequencies using amplicon sequencing data. All sequencing reads were first aligned to the reference sequence using BWA (v0.7.17) [21]. Samtools (v1.9) was subsequently used for file format conversion and sorting, generating standardized input files for downstream analyses [22].

Following alignment and basic counting procedures, mutation data were further integrated and statistically analyzed in R (version 4.4.1). This analysis included matching sequencing counts to the theoretically designed mutation set, calculating the relative abundance of each variant under different experimental conditions, and performing normalization and quality-control steps to evaluate library coverage and data reliability. These processed datasets provided the foundation for subsequent escape analyses.

#### 2.10.2. Validation of Plasmid Backbone Integrity (Tn5 Sequencing)

After completion of the standard alignment workflow, Tn5 sequencing data were visualized using IGV (v2.8.0) [23]. Coverage uniformity across the full-length plasmid backbone, read depth distribution, and potential structural variation signals were carefully examined to exclude large-scale deletions or rearrangements that might have been introduced during the cloning process.

#### 2.10.3. Quantification of Mutation Frequencies (Amplicon Sequencing)

For mutation frequency quantification, amplicon sequencing data were processed following the standard alignment workflow. Raw paired-end reads were first merged using bbmerge (v38.84) [24]. Adapter and primer sequences were then trimmed using Cutadapt (v3.5) to obtain high-quality target fragments [25]. The pre-processed sequences were subsequently clustered and counted using Starcode (v1.3), generating a count matrix of unique variants for downstream quantitative analysis [26].

#### 2.10.4. Variant Count Statistics and Analysis

An R-based analysis workflow was implemented to integrate theoretically designed mutations with sequencing counts and calculate the relative frequencies and distribution characteristics of each variant. First, a single-codon substitution variant set was generated at the codon level based on the reference sequence, while retaining the wild-type sequence as a control. Sequencing count files obtained after clustering were then imported, and the observed sequences were matched and merged with the designed variant set. Entries with lengths or sequence structures inconsistent with the theoretical design were removed to ensure that subsequent analyses were based only on the variant set that conformed to the designed specifications. Designed variants that were not detected were assigned a count of zero to maintain consistency between the designed set and the observed data. We applied a minimum frequency threshold to filter variants and reduce statistical noise introduced by low-abundance variants. Normalized frequencies were then calculated using the filtered designed variant set as the denominator, enabling comparisons across experimental conditions. Finally, the data were organized into matrix format by mutation site and amino acid substitution, and library coverage and mutational enrichment patterns were systematically evaluated based on the frequency distributions.

#### 2.10.5. Calculation of Serum Escape Mediated by Spike RBD Mutations

In the neutralization escape assay of breakthrough infection serum, the escape metric was calculated following a previously published method [20]. For each variant (*v*) at serum at its pre-determined dilution (*c*), the raw escape enrichment ratio, *pv*(*c*), was calculated as follows:

$$p_{v}\left(c\right)=\frac{f_{v,\mathrm{Spike},c}/f_{v,\mathrm{VSV}-G,c}}{f_{v,\mathrm{Spike},0}/f_{v,\mathrm{VSV}-G,0}}$$

where *fv*,*Spike*,*c* and *fv,VSV-G,c* represent the sequencing frequencies of variant v at serum dilution c in the spike-pseudotyped library (infecting HEK-293T-ACE2 cells) and the VSV-G-pseudotyped library (infecting HEK-293T cells), respectively. *fv*,*Spike*,*0* and *fv*,*VSV-G*,*0* denote the corresponding sequencing frequencies under the no-serum control condition. To avoid undefined values resulting from zero counts, a small pseudocount of 10^−8^ was added uniformly to all counts before calculation. The raw *pv*(*c*) value represents a ratio of relative enrichment factors rather than a probability and is therefore not intrinsically bounded between 0 and 1. To facilitate visualization and comparison across mutations, the raw values were normalized by the global maximum:

$$P_{v}\left(c\right)=\frac{p_{v}\left(c\right)}{{max}_{v,c}/{[p}_{v}\left(c\right)]}$$

Because all raw (*pv*(*c*)) values are non-negative and the denominator corresponds to the global maximum value, the normalized escape value (*Pv*(*c*)) ranges from 0 to 1. The normalized values were subsequently used for two types of visualization. At the site level, the normalized escape values of all amino acid substitutions at the same site were summed to obtain the cumulative escape score for that site. A site-level escape score of 0.1 was used as an operational threshold to distinguish relatively high from relatively low cumulative escape signals. This threshold was determined based on the overall distribution of the normalized escape scores and was used primarily for result stratification and visualization, allowing the separation of a small number of discrete higher peaks from broadly distributed low-level background signals. This threshold does not correspond to a specific fold change in IC_50_ or to a clinically defined neutralization cutoff.

Biological replicates were performed for each serum sample. Specifically, the same mutant library plasmid was used for both replicates, while all subsequent steps, including screening of the genotype-phenotype linked cell library, pseudovirus preparation, screening by serum IC_80_, HEK-293T-ACE2 cell infection, genomic DNA extraction, sequencing library preparation and sequencing were carried out independently. Thus, biological replicates reflected independent experimental processing from viral library production and serum selection through sequencing. For DMS escape analysis, normalized escape scores from biological replicates were averaged after quality control. Unless otherwise specified, replicate-derived values are presented as the mean of independent biological replicates. Mutations with insufficient read counts or unreliable representation after filtering were excluded from quantitative interpretation.

### 2.11. Collection and Processing of Real-World SARS-CoV-2 Variant Sequences

Real-world circulating variant sequences were obtained from SARS-CoV-2 genomic surveillance conducted in Jiangsu Province in 2024 and 2025. Samples included in the analysis were derived from individuals confirmed positive by nucleic acid testing and met sequencing quality control criteria, including sufficient viral load and appropriate sample preservation conditions. Following high-throughput sequencing, raw data were processed according to established quality control pipelines. Only viral whole-genome sequences meeting coverage thresholds and lacking large deletions or assembly abnormalities were retained for downstream analysis.

To generate standardized spike protein sequences, the collected SARS-CoV-2 whole-genome sequences were first processed using the Wuhan-Hu-1 reference genome as the framework. The boundaries of the spike coding region were defined based on the reference annotation, and the corresponding spike gene sequences were extracted from each genome. Extracted spike sequences were aligned together with the Wuhan-Hu-1 spike reference sequence using reference-guided multiple sequence alignment implemented in MAFFT to standardize sequence length and reading frame position. After alignment, gaps introduced during alignment were removed, and sequences were trimmed to lengths divisible by three to avoid frameshift errors. Translation was performed according to the standard genetic code, yielding a set of reference-guided full-length spike amino acid sequences. At the amino acid level, extraction of the RBD region was performed using a combination of conserved motif matching and sequence similarity-based localization. The Wuhan-Hu-1 RBD boundary motifs (RVQPTE at the N-terminus and NKCVNF at the C-terminus) were used as reference anchors. Exact motif matching was prioritized to determine RBD start and end positions. When amino acid substitutions were present within these boundary regions in certain variants, local sequence similarity alignment was applied to identify the optimal matching segment, ensuring accurate localization of the RBD despite local variation. Only sequences with successfully identified RBD boundaries and lengths within a biologically reasonable range were retained, excluding sequences with abnormal translation or incomplete coverage. The standardized RBD FASTA dataset was ultimately generated for integrated analysis of real-world surveillance variants and laboratory DMS serum escape maps.

For comparisons between DMS-identified escape mutations and mutations observed in real-world SARS-CoV-2 surveillance sequences, overlap enrichment was evaluated using Fisher’s exact test. The background set was defined as all designed single amino acid substitutions within the RBD library that passed quality-control filtering. All statistical analyses were performed using R (version 4.4.1) and Python (version 3.10).

## 3. Results

### 3.1. Establishment of a Genotype-Phenotype Linked DMS Platform for Serum Escape Analysis

Building on a previously established pseudovirus-based DMS approach [19], we adapted the core experimental framework and applied it to DMS-based serum escape profiling of the JN.1 and XEC spike RBD (residues 319–541). This system strictly maintains a one-to-one genotype–phenotype linkage, ensuring each cell expresses only a single mutant. Consequently, it enables precise quantitative assessment of serum escape for individual variants (Figure S1). Quality assessment of the JN.1 and XEC mutant libraries integrated into HEK-293T-rtTA cells demonstrated that, at a sequencing depth of approximately 106 reads, the vast majority of designed single-codon substitutions were successfully detected (>99%) in both variant backgrounds, indicating high library coverage and highly similar mutational distributions between the two variant libraries. The frequency distributions across different amino acid positions and substitution types were generally uniform, with enrichment observed at only a limited number of sites, likely resulting from oligonucleotide synthesis bias. Overall, the frequencies of individual single-codon mutants were primarily distributed at the order of 10^−4^, while the cumulative mutation frequencies at most residue positions were approximately 0.02, with only a small number of sites reaching higher values (up to approximately 0.06). When aggregated across all positions, the total frequencies of most substitution types remained below 0.1, with only a small subset approaching 0.15 (Figure S2).

We performed serum escape screening using 10 serum samples collected from participants who had experienced breakthrough infections. All participants had received three doses of inactivated SARS-CoV-2 vaccine (CoronaVac) and subsequently experienced a BA.4/5-associated breakthrough infection between 7 December 2022 and 31 January 2023. Serum samples were collected seven months after the breakthrough infection (Table 1).

**Figure 1:**
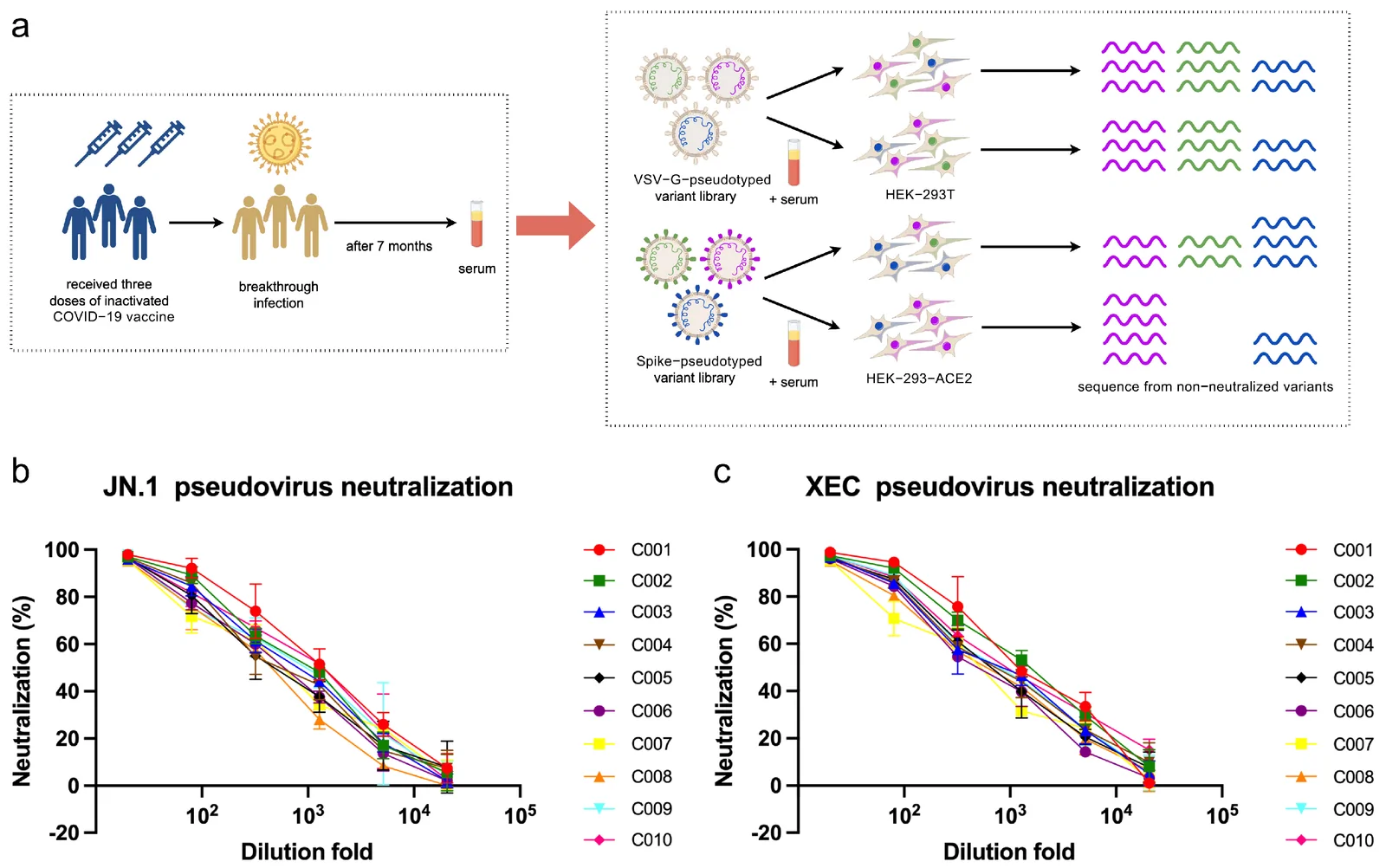
Serum immune evasion DMS workflow and serum dilution fold determination. (**a**) The DMS system used to detect serum escape. Serum samples (*n* = 10) were collected seven months after breakthrough infection from subjects who had received three doses of inactivated COVID-19 vaccines. A VSV-G or spike protein pseudovirus library was incubated with predetermined concentrations of serum, while the control group received no serum. These mixtures were then used to infect HEK-293T or HEK-293-ACE2 cells. Cells were collected post-infection with or without serum, and the sequences of infected variants were detected by high-throughput sequencing. This allowed for a quantitative assessment of the extent to which variants escaped serum antibody neutralization. Pseudovirus neutralization assay was performed using pseudoviruses carrying the JN.1 (**b**) and XEC (**c**) spike proteins to determine the serum concentrations used in the serum escape assay. Error bars represent the standard error between two technical replicates.

Using the constructed JN.1 and XEC spike RBD mutant libraries, we performed serum escape assays in a pseudovirus system and quantified changes in variant frequencies by high-throughput sequencing (Figure 1a). Before the formal DMS-based serum selection, we conducted standard pseudovirus neutralization assays for each serum sample against both JN.1 and XEC variants to determine the appropriate selection pressure (Figure 1b,c). Based on the neutralization potency of each serum, we selected the dilution corresponding to the IC80 value as the criterion for DMS screening. Specifically, for the JN.1 variant, the IC80 dilution titers of the 10 sera were as follows: C001 (1:243.7); C002 (1:156.3); C003 (1:121.0); C004 (1:99.6); C005 (1:80.8); C006 (1:90.7); C007 (1:66.7); C008 (1:80.8); C009 (1:94.7) and C010 (1:147.0). For the XEC variant, the corresponding IC80 dilution titers were: C001 (1:269.8); C002 (1:214.4); C003 (1:109.8); C004 (1:107.3); C005 (1:117.3); C006 (1:96.1); C007 (1:61.1); C008 (1:83.2); C009 (1:127.3) and C010 (1:124.2).

### 3.2. Serum-Specific Escape Landscapes of JN.1 and XEC RBD Mutations in the Context of Breakthrough Infections

**Figure 2:**
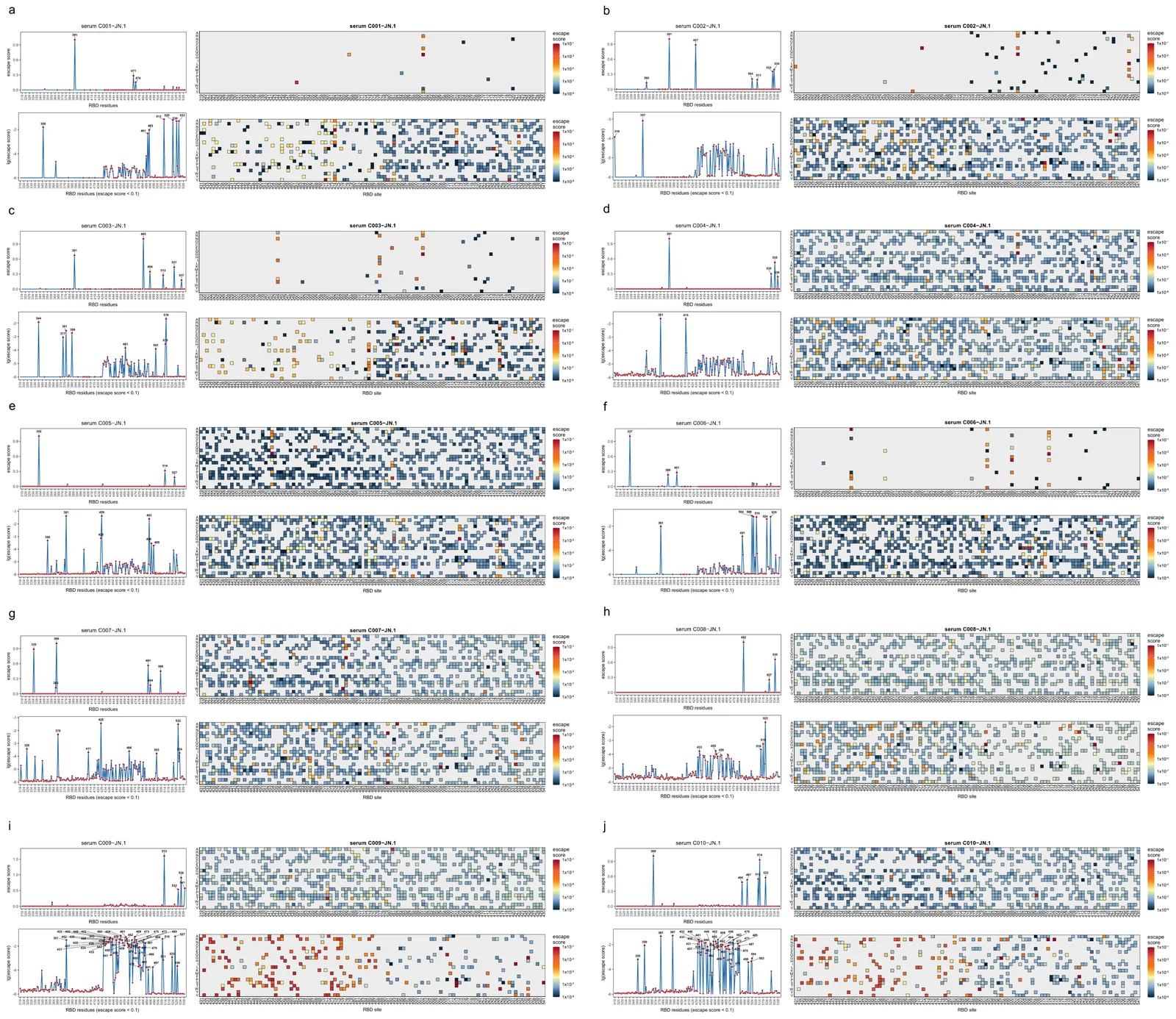
Serum antibody escape JN.1 spike RBD mutation landscape in individuals with breakthrough infection. Serum antibody escape effects at each site in the JN.1 spike protein RBD (residues 319–541). Serum samples were obtained from individuals who had received three doses of inactivated COVID-19 vaccine and subsequently experienced breakthrough infection, with samples collected 7 months after infection. Escape effects were calculated for each of the 10 sera (**a**–**j**) and averaged across individuals. The left line plots show the total positive escape effect accumulated from all amino acid mutations at each RBD site. The upper panel displays the overall escape landscape, whereas the lower panel highlights regions with low escape (escape score < 0.1, log10 scale). The right heatmaps show the relative escape effect caused by single amino acid substitutions (log10 scale), with RBD sites on the x-axis and substituted amino acids on the y-axis. Gray areas indicate that no escape effect was detected for that mutation. Normalized escape scores reflect relative mutation enrichment and do not represent absolute escape probabilities or specific fold reductions in neutralizing-antibody titers or IC_50_ values.

Comparison across the 10 breakthrough infection sera revealed pronounced site specificity and variability in the neutralization sensitivity profiles of JN.1 spike RBD mutations (Figure 2). Overall, mutations at most RBD sites induced only modest reductions in neutralizing activity. At the single amino acid substitution level, the majority of mutations exhibited escape scores ranging from approximately 10^−6^ to 10^−8^, indicating generally limited effects on serum neutralization. After integrating the escape effects of all amino acid substitutions at each site, only a small number of sites accumulated high-intensity escape signals (escape score > 0.1), which were typically restricted to single residues or small clusters of adjacent positions. Meanwhile, substantial differences in both the intensity and spatial distribution of escape signals were observed among sera. For ease of result stratification and description, a site-level escape score > 0.1 was defined as a relatively high cumulative escape signal. It should be noted that the escape score is a normalized relative-enrichment metric intended for comparison among mutations. It does not represent an absolute probability of immune escape or a specific fold reduction in neutralizing-antibody titer. Therefore, 0.1 is a descriptive stratification threshold rather than corresponding to a fixed fold change in IC_50_.

Across the 4659 designed amino-acid-level categories, 222 stop-codon substitutions were excluded, leaving 4437 non-stop amino acid substitutions for quantifiability assessment. After quality control, 2717–2742 substitutions per serum sample were reliably quantified in the JN.1 background, corresponding to a median quantifiable proportion of 61.58% (range, 61.24–61.80%). In the XEC background, 2664–2698 substitutions were reliably quantified, corresponding to a median proportion of 60.43% (range, 60.04–60.81%).

The site-level escape score represented the cumulative escape effects of all amino acid substitutions at a given residue. Based on this analysis, several sites repeatedly exhibited strong site-level escape effects across multiple sera, including residue 391 and multiple residues within the C-terminal region of the RBD (approximately residues 470–510). After integrating the escape effects of all substitutions at these sites, these regions exhibited higher cumulative escape signals (escape score > 0.1), suggesting that they may be subjected to substantial antibody selection pressure under breakthrough infection-induced immune backgrounds. In contrast, within low-level cumulative escape regions at the site level (escape score < 0.1), multiple RBD segments displayed continuous low-to-moderate cumulative escape signals composed of adjacent residues (log10(escape score) > −5), a pattern that was particularly evident within the receptor-binding motif (RBM). Despite the presence of continuous cumulative escape signals across neighboring sites, their overall cumulative effects remained limited and failed to form broad higher-intensity cumulative escape regions at the site level (escape score > 0.1).

Combining analyses of escape sites and individual amino acid substitutions further revealed pronounced differences in both the spatial distribution and intensity of escape signals across sera. In some sera (such as C001, C002, C003, and C006), escape signals were mainly concentrated at a limited number of sites, predominantly downstream of residue 430, and were characterized by the coexistence of a small number of high-intensity mutations (escape score > 10^−3^) and many low-level mutations (escape score < 10^−7^). In contrast, other sera (C004, C005, C007, C008, C009, and C010) exhibited more broadly distributed escape signals across the RBD, although the overall signals were still dominated by low-intensity mutations (escape score < 10^−7^). Notably, although sera C009 and C010 displayed relatively limited site-level cumulative escape effects within the 430–490 region (escape score < 0.1), many individual amino acid substitutions within this region exhibited high-intensity escape effects (escape score > 10^−3^). This finding suggests that strong escape effects from individual mutations may be diluted at the site level by their overall distribution, thereby failing to generate prominent site-level higher-intensity cumulative escape signals.

**Figure 3:**
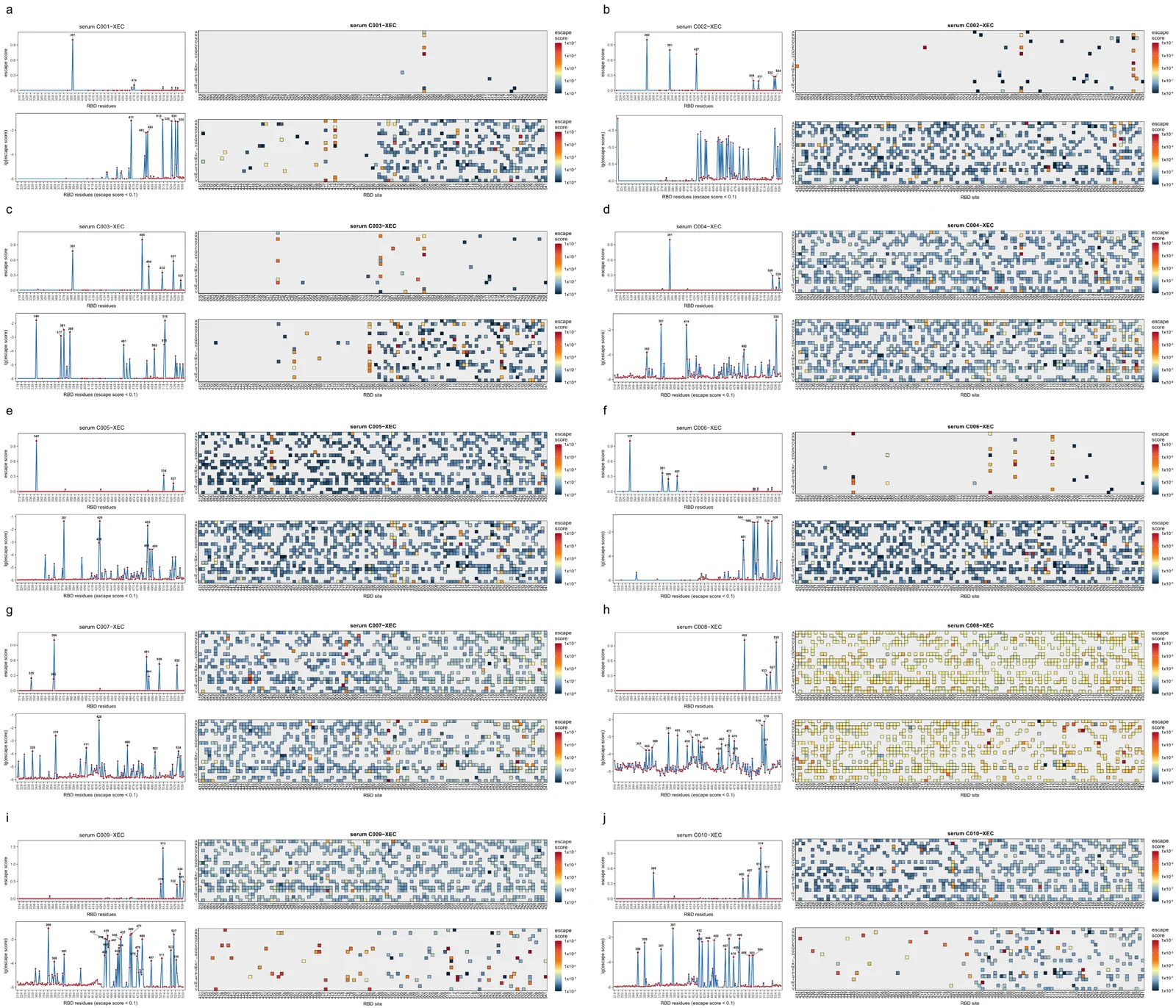
Serum antibody escape XEC spike RBD mutation landscape in individuals with breakthrough infection. Serum antibody escape effects at each site in the XEC spike protein RBD (residues 319–541). Serum samples were obtained from individuals who had received three doses of inactivated COVID-19 vaccine and subsequently experienced breakthrough infection, with samples collected 7 months after infection. Escape effects were calculated for each of the 10 sera (**a**–**j**) and averaged across individuals. The left line plots show the total positive escape effect accumulated from all amino acid mutations at each RBD site. The upper panel displays the overall escape landscape, whereas the lower panel highlights regions with low escape (escape score < 0.1, log10 scale). The right heatmaps show the relative escape effect caused by single amino acid substitutions (log10 scale), with RBD sites on the x-axis and substituted amino acids on the y-axis. Gray areas indicate that no escape effect was detected for that mutation. Normalized escape scores reflect relative mutation enrichment and do not represent absolute escape probabilities or specific fold reductions in neutralizing-antibody titers or IC_50_ values.

Escape profiling of XEC spike RBD mutations similarly demonstrated marked inter-serum heterogeneity (Figure 3). At the site level, the escape sites identified for JN.1 and XEC in the same serum background exhibited high overlap, with an average overlap ratio of approximately 89.79%. At the individual amino acid substitution level, the escape score distributions of the two variants in the same serum background also exhibited comparable median levels, with an average median difference of approximately 2.41-fold and no significant overall difference observed (paired Wilcoxon signed-rank test, *p* = 0.760). Overall, most single amino acid substitutions still exerted only limited effects on neutralization (escape score < 10^−6^), whereas higher-intensity escape signals remained concentrated at a limited number of key residues after site-level accumulation.

However, compared with JN.1, the major differences observed for XEC at the site level were primarily confined to low-level cumulative escape regions (escape score < 0.1). Specifically, relative to the more continuous distribution pattern of low-to-moderate cumulative escape signals observed in JN.1, XEC displayed weakened or discontinuous cumulative escape signals in several sera. In sera C001, C003, and C006, low-level cumulative escape signals within the RBM were markedly reduced in XEC compared with JN.1, decreasing by approximately 100-fold in C001 and C003, and by approximately 10-fold in C006. Furthermore, in sera C001 and C003, multiple sites within the 430–480 region exhibited no detectable escape effects in XEC, resulting in a substantially sparser distribution of escape sites. Correspondingly, the numbers of escape sites decreased from 112 and 120 in JN.1 to 79 and 82 in XEC, respectively. In contrast, although serum C008 exhibited largely similar higher-intensity cumulative escape sites (escape score > 0.1) between the two variants, the overall escape signal intensity at the individual amino acid substitution level (escape score > 10^−6^) was approximately 10-fold higher in XEC than in JN.1.

Despite these differences in low-intensity escape structures, correlation analysis demonstrated that escape scores of corresponding amino acid substitutions remained highly consistent between JN.1 and XEC within the same serum background, with an average correlation coefficient of approximately 0.823 across the 10 sera (Figure S3). These findings suggest that the major escape pathways of the two variants remain broadly shared under serum immune pressure. The standard pseudovirus neutralization assay further validated the DMS results. Compared with pseudoviruses bearing the wild-type spike protein, pseudoviruses carrying representative escape mutations exhibited reduced neutralization sensitivity, with effect directions consistent with those identified by the DMS (Figure S4).

### 3.3. Widespread Distribution and Mutational Complexity of Serum Escape Sites Across the RBD

We evaluated the overall prevalence of escape potential by calculating the proportion of RBD residues classified as escape or non-escape sites in JN.1 and XEC (Figure S5). Here, escape amino-acid substitutions were defined as mutations that exhibited measurable serum escape effects in at least five of the tested sera. Escape sites were further defined as RBD residues harboring five or more distinct escape-permissive substitutions. Based on these criteria, we systematically quantified the distribution and characteristics of escape sites across the RBD of JN.1 and XEC variants. The results showed that escape sites accounted for 78% and 62% of RBD residues in JN.1 and XEC, respectively (Figure S5a,b), indicating that under the current population immune background shaped by vaccination and breakthrough infection, a large proportion of RBD residues still retain the potential to mediate serum escape through mutation.

However, the proportion of escape sites alone does not reflect the mutational plasticity of these residues or the diversity of evolutionary pathways leading to immune evasion. Therefore, we also examined the mutational complexity of escape sites, defined as the number of distinct amino-acid substitutions at a specific residue capable of escaping serum neutralization (Figure S6). In both variants, most escape sites could accommodate multiple escape substitutions. In JN.1, sites harboring six and seven distinct escape substitutions accounted for 42% and 15% of all escape sites, respectively, while in XEC the corresponding proportions were 46% and 9% (Figure S6a,d). Nevertheless, no position was observed to support more than seven escape substitutions, suggesting that mutational flexibility at these positions remains constrained. The RBM accounts for approximately 31% of the full-length RBD. In JN.1 and XEC, the corresponding proportions of escape sites located within the RBM are 32% and 34%, respectively, roughly matching the RBM’s relative length. Notably, the majority of escape sites (68% and 66% in JN.1 and XEC, respectively) are located outside the RBM (Figure S6b–e).

Further stratification by mutational complexity revealed that non-RBM escape sites consistently remained predominant across all complexity levels (each accounting for more than 50%), and the enrichment of escape sites within the RBM did not exhibit a consistent trend with increasing mutational complexity (Figure S6c–f).

### 3.4. Temporal Dynamics of SARS-CoV-2 Epidemic Variant Lineages and Correspondence with Serum Escape Mutations

**Figure 4:**
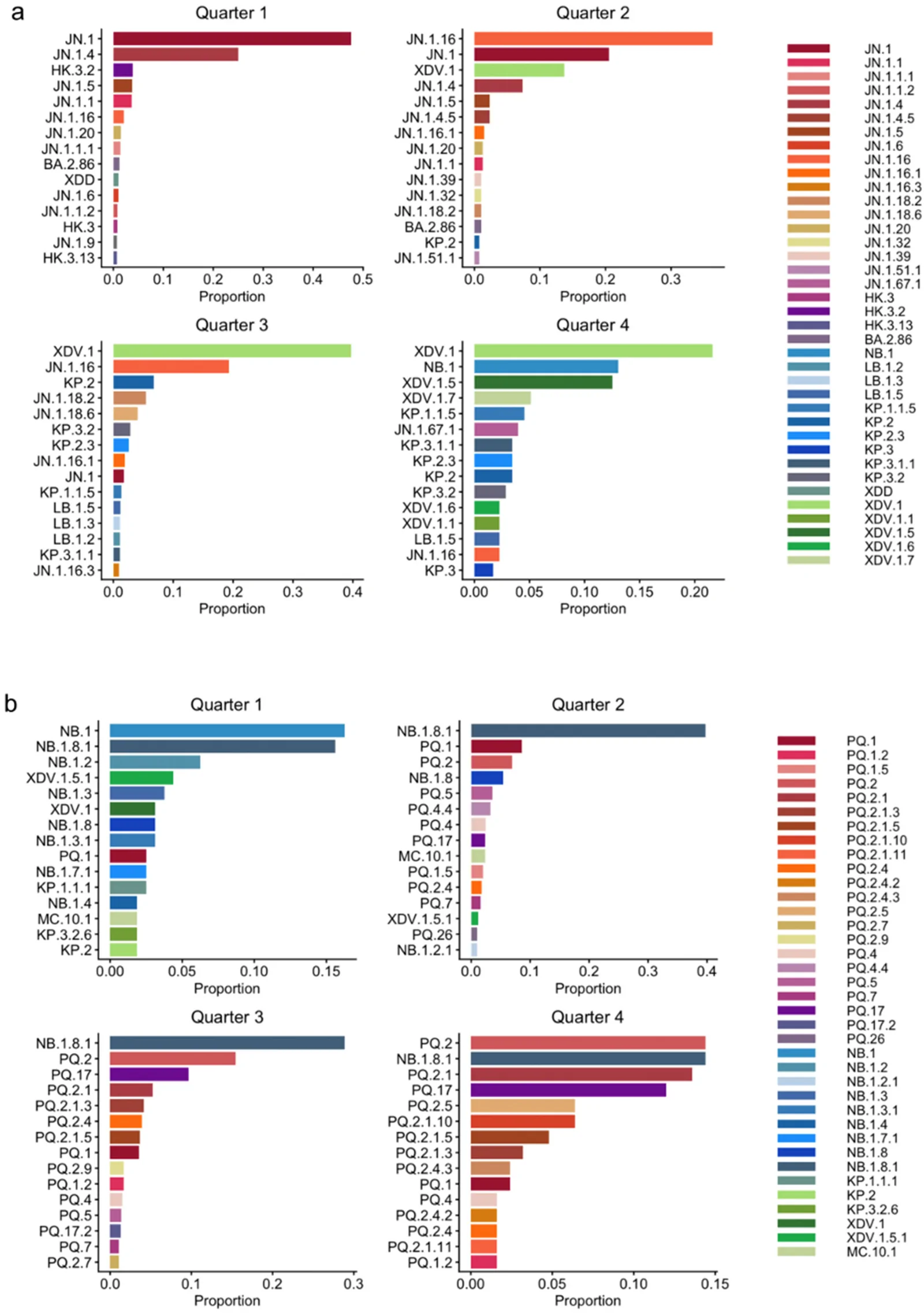
Temporal distribution of epidemic SARS-CoV-2 lineages in 2024 and 2025 based on genomic surveillance. Distribution of SARS-CoV-2 lineages identified through genomic surveillance across the four quarters of 2024 (**a**) and 2025 (**b**). Each panel shows the proportion assigned to major lineages in each quarter (Quarter 1–Quarter 4). Only the top 15 most frequently detected lineages are displayed. Bars represent the proportion of sequences per lineage, and colors indicate distinct lineage classifications, which are consistently applied across each panel to enable comparison over time.

We analyzed genomic surveillance data of SARS-CoV-2 infections in Jiangsu Province across four quarters of 2024 and 2025 to characterize dynamic changes in epidemic variant lineages (Figure 4). The results showed that JN.1 and its sublineages predominated during the first two quarters of 2024, followed by a gradual transition toward emerging lineages, including XDV and KP lineages, indicating a continuous process of lineage replacement over time. Compared with 2024, the lineage composition in 2025 exhibited marked differences. The proportion of XDV-related lineages declined, whereas NB.1 and its sublineages (particularly NB.1.8.1) gradually became the predominant circulating lineages. Meanwhile, the PQ lineage continuously expanded throughout the year and eventually became the dominant epidemic variant in the later period. Notably, both PQ and NB lineages originated from XDV-related branches and further accumulated key mutations on this genetic background, giving rise to new lineages with transmission advantages.

**Figure 5:**
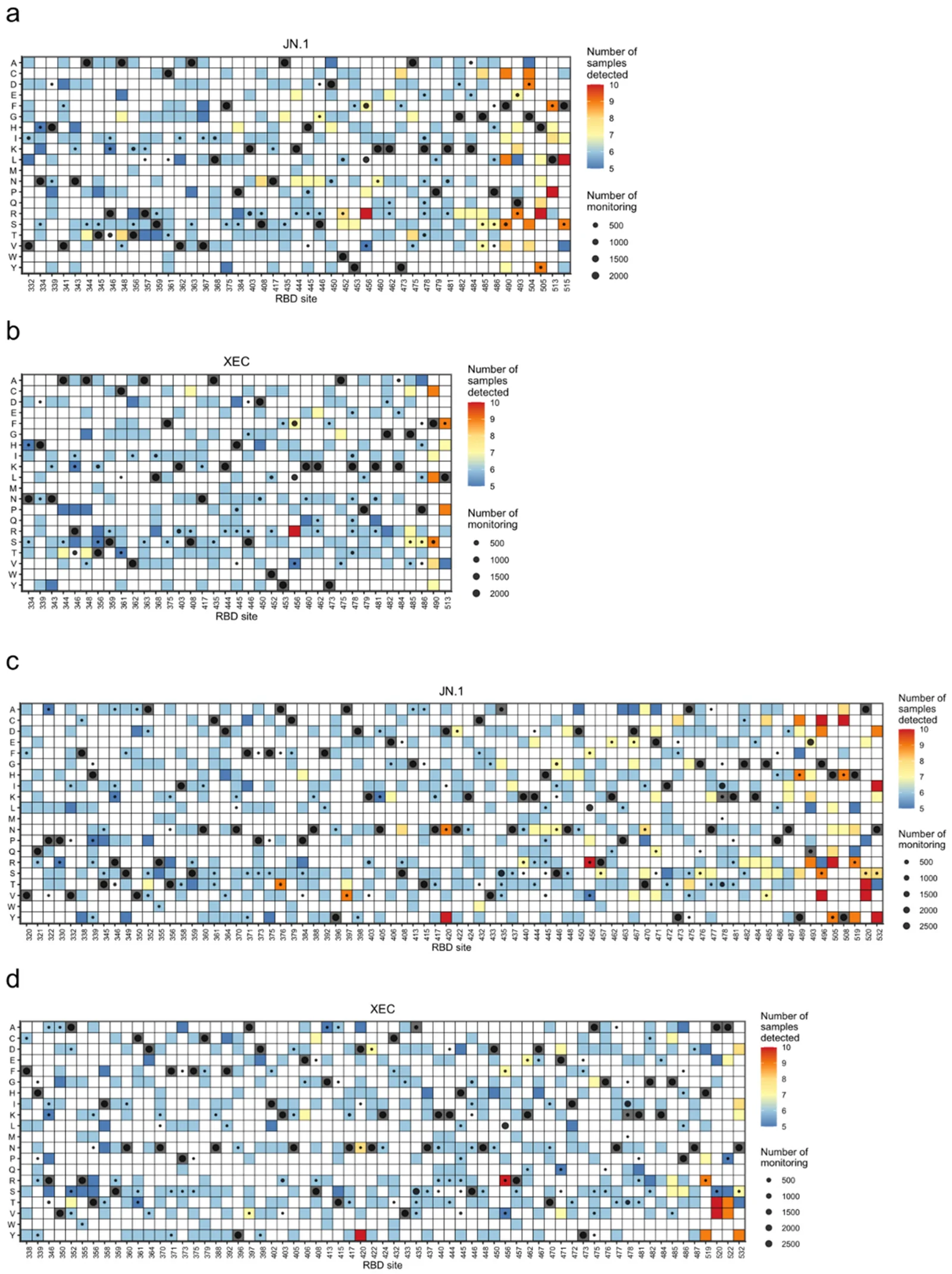
Comparison of the distribution of RBD serum escape mutations in JN.1 and XEC with mutations detected in epidemic variants during 2024 and 2025. These comparisons highlight sites and mutations of overlap between experimentally defined JN.1 (**a**,**c**) and XEC (**b**,**d**) RBD mutations escape breakthrough infection sera and those present in the epidemic variants of SARS-CoV-2 infection surveillance in Jiangsu Province in 2024 (**a**,**b**) and 2025 (**c**,**d**), respectively. The x-axis indicates RBD residue positions, and the y-axis represents amino acid substitutions. Each square corresponds to a single amino acid substitution at RBD site. Colored squares denote mutations identified as serum escape substitutions by DMS, with different colors reflecting the number of breakthrough infection sera (out of 10 tested samples) in which escape was detected. Black dots indicate mutations observed in variants from epidemiological surveillance. The size of the black dot represents the number of occurrences of mutations at each site among epidemic variants identified in the 2024 or 2025 Jiangsu Province SARS-CoV-2 surveillance dataset. Grey squares represent the reference amino acids at each RBD position of JN.1 and XEC.

We systematically compared RBD escape sites and substitutions identified by DMS with mutations observed in genomic surveillance data from 2024 and 2025 (Figure 5 and Figure S7). In both JN.1 and XEC, a significant overlap was observed between DMS-identified escape sites and mutations detected in circulating variants, and this overlap was markedly enhanced in 2025 (Fisher’s exact test, *p* < 0.001): site overlap rates for JN.1 and XEC rose from 27.17% and 26.81% to 45.09% and 47.10%, respectively. A similar trend was observed at the substitution level, with overlap rates increasing from 6.58% and 6.94% to 10.73% and 11.70%. Spatially, the overlapping signals in 2025 further expanded into non-RBM regions.

At the individual site level, the degree of overlap varied across RBD positions. Some sites simultaneously exhibit strong escape signals and high observed mutation frequencies (e.g., sites 346, 445, 446, 456, and 478 in JN.1; sites 346, 445, 478, and 486 in XEC). In contrast, certain sites exhibited strong escape effects but relatively low mutation frequencies in epidemic variants (e.g., sites 513 and 515 in JN.1; sites 490 and 513 in XEC). Additionally, some sites showed both weak escape signals and low frequency of surveillance mutations (e.g., sites 332, 345, and 358 in JN.1; sites 359, 362, and 412 in XEC).

### 3.5. Comparison with the Ancestral Antigenic Background Reveals Distribution Patterns and Reversion of Escape Mutations

**Figure 6:**
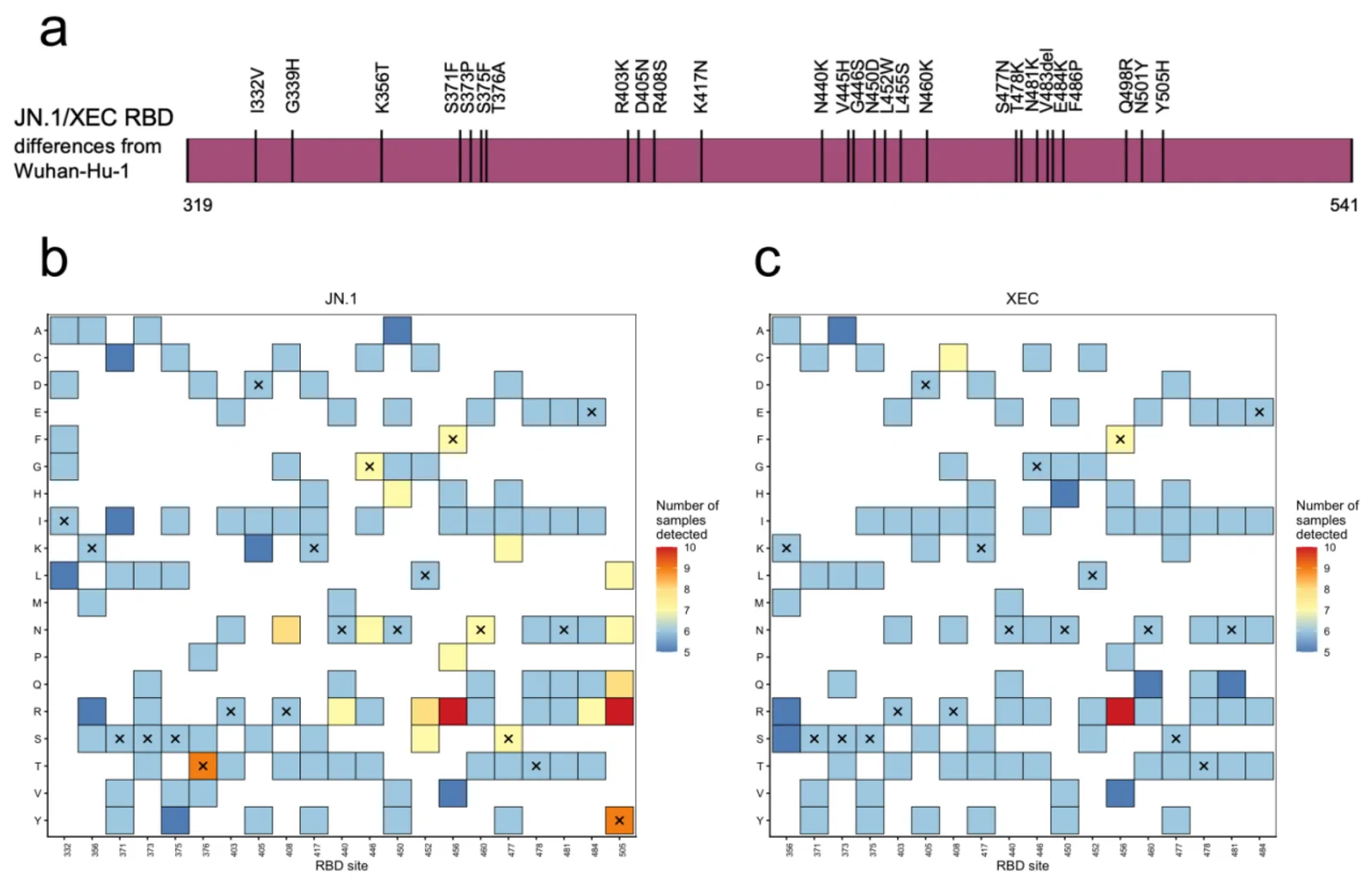
Comparison of amino acid identity between JN.1 and XEC RBD serum escape mutations and SARS-CoV-2 ancestral strain (Wuhan-Hu-1). (**a**) Diagram of the RBD substitutions that distinguish JN.1 and XEC from SARS-CoV-2 ancestral strain (Wuhan-Hu-1). DMS serum escape maps, corresponding to the RBD of JN.1 (**b**) and XEC (**c**), respectively. The x-axis represents the RBD residue position, and the y-axis represents the amino acid substitution. Each square corresponds to a single amino acid substitution at a specific site. Different colors represent the number of samples in the breakthrough infection serum (10 test samples) where the substitution resulted in detectable escape. The “×” indicates the reference amino acid at each position in the RBD of the ancestral SARS-CoV-2 strain (Wuhan-Hu-1).

Wuhan-Hu-1 (GenBank: MN908947) served as the foundation for early vaccine design and population immune memory. Therefore, comparing escape mutations with this ancestral antigenic background helps to understand the potential evolutionary trajectories of the virus. We analyzed DMS-identified escape substitutions in JN.1 and XEC and compared them with the Wuhan-Hu-1 RBD sequence (Figure 6). Amino acid differences between both variants and Wuhan-Hu-1 were widely distributed across the RBD and exhibited clear clustering in functionally important regions, such as sites 371–376 and 477–486 (Figure 6a). Notably, at certain escape sites, the identified escape substitutions were identical to the corresponding amino acids in Wuhan-Hu-1 (Figure 6b,c). This phenomenon-reversion to ancestral residues was observed in both variants and occurred across multiple RBD sites.

Further analysis revealed that these sites often harbored multiple forms of escape substitutions, including both substitutions identical to and different from the ancestral amino acids, suggesting a high degree of mutational flexibility. Most escape sites involving reversion to ancestral residues were shared between the two variants, although a small number of lineage-specific sites were also observed.

## 4. Discussion

This study reveals the hierarchical features of the serum escape landscape of SARS-CoV-2 under the immune pressure of a specific hybrid-immune background induced by three-dose inactivated-vaccine immunization followed by BA.4/5 breakthrough infection, characterized by broadly distributed low-magnitude escape signals with focal enhancement at limited key sites, rather than isolated dominant epitopes within the RBM. This pattern indicates serum antibody recognition targets multiple spatially adjacent or functionally linked residues. Meanwhile, receptor-binding constraints restrict substitutions within the RBM to mild, tunable escape effects instead of potent single mutations [13,14]. Consistent with prior polyclonal serum DMS from Bloom et al., this dispersed, low-magnitude escape pattern represents an extended polyclonal serum escape profile specific to late Omicron variants, jointly shaped by viral genetic background, immune exposure history and serum antibody composition [27,28,29].

Distinct escape profiles across different pooled serum samples reflected substantial heterogeneity in post-breakthrough humoral responses in both epitope targeting and neutralization potency. Sera C001, C002, C003, C006, C009 and C010 exhibited unevenly concentrated escape peaks, while C004, C005, C007 and C008 generated moderate signals across broader RBD regions, corresponding to dispersed epitope recognition. Such inter-individual divergence stems from varied immune exposure, memory B cell repertoires and clonally expanded dominant antibody clones [2,15,16,17,18]. Under this composite immune landscape, single substitutions rarely trigger population-level neutralization escape, and cumulative moderate-effect mutations across multiple residues jointly drive functional immune evasion [2]. This inter-serum difference is observed in the context of both the JN.1 and XEC variants, consistent with the results of previous polyclonal serum DMS studies [7,27,28,29].

From a broader perspective of population immunity evolution, in the early stages of the COVID-19 pandemic, the population generally lacked prior immunity against SARS-CoV-2. However, with widespread vaccination and multiple rounds of natural infection, population immune status has gradually shifted toward hybrid immunity formed by a combination of vaccine immunity and infection immunity. Compared to single vaccination or natural infection, hybrid immunity generally elicits a broader spectrum of antibody recognition and immune memory, and markedly reduces the risks of severe disease, hospitalization and death, thereby reshaping the clinical manifestations and public health impacts of COVID-19. However, immune protection wanes over time, and continuous viral antigenic evolution together with immune escape means that hybrid immunity cannot completely block reinfection and virus transmission. Against the backdrop of widespread pre-existing population immunity, current priorities for COVID-19 prevention and control have gradually shifted toward continuous surveillance of viral evolution, identifying the risk of immune escape, and targeted protection of high-risk groups. The serum samples used in this study, obtained after three-dose inactivated vaccine immunization plus BA.4/5 breakthrough infection, represent the typical immune background after the immune shift in this population. The dispersed, low-intensity escape signals we observed reveal potential adaptive strategies employed by SARS-CoV-2 under the pressure of established hybrid immunity [30].

Within this overall immune context, JN.1 and XEC displayed high concordance at major high-intensity escape hotspots, proving core antigenic determinants remain stable during recent viral evolution. Given their closely related RBD sequences, their cumulative escape region spanning residues 430–510 largely overlaps with the primary serum escape zone reported in Bloom laboratory’s KP.3.1.1 DMS research, which identified residues 475, 478, 487 and 505 as dominant immunodominant targets for JN.1-exposed sera. Over the past two years, circulating variants such as XFG, NB.1.8.1 and LP.8.1 have continuously acquired amino-acid substitutions within the 470–510 region, which is consistent with the interpretation that these sites are subject to population antibody-mediated selective pressure [29]. Despite shared major escape regions, direct quantitative comparison of escape scores between the two studies is invalid due to divergent experimental systems, participant cohorts and bioinformatic pipelines. First, genetic background-dependent epistatic effects alter the escape phenotype of identical amino acid substitutions: mutations such as A435S and E493D/N exert opposing functional impacts across distinct spike backbones. Second, serum donors had fundamentally different immune imprinting: Bloom’s cohort received ancestral mRNA vaccines followed by JN.1 vaccination and natural infection, whereas our participants only obtained three-dose inactivated ancestral vaccines and BA.4/5 breakthrough infection without prior JN.1 exposure, reshaping neutralizing immunodominance hierarchies and leading to only low-level escape signals at 475, 478, 487 and 505 in our dataset [29].

Although core escape hotspots are conserved between the two variants, XEC showed greater inter-serum variability in low-magnitude escape signals, with attenuated contiguous mild escape traces within the RBM in several serum groups. These strain-specific differences arose from systematic redistribution of pre-existing escape effects rather than newly generated high-impact escape sites, revealing a critical adaptive strategy of SARS-CoV-2 under complex population immunity: The virus may expand its capacity for immune adaptation by fine-tuning the effects of multiple moderate-impact mutations without disrupting core functional regions. Genetic background-dependent epistatic effects reported in previous studies may partly explain these phenotypic differences [29].

Beyond mapping escape hotspots, quantification of escape-permissive substitutions at each residue provides a more accurate metric for evolutionary potential than raw mutation frequency. Escape sites spread broadly across the full RBD without specific enrichment within the RBM, contradicting the canonical view that the RBM serves as the sole primary antigenic hotspot. Non-RBM residues, despite lacking core neutralizing antibody interfaces, indirectly disrupt antibody recognition via shifts in local conformation, surface charge, glycosylation and surrounding epitope microenvironments [3,31,32,33]. These findings revise existing models of Omicron-era antigenic evolution by demonstrating that viral escape from polyclonal post-breakthrough immunity is not limited to direct interference with receptor-binding epitopes.

Strong consistency between our in vitro DMS data and 2024–2025 Jiangsu provincial genomic surveillance data delivers critical public health implications. The overlap between experimentally identified escape mutations and mutations carried by circulating variants increased markedly in 2025. Compared with the relatively restricted mutation distribution observed in 2024, circulating mutations in 2025 were more broadly distributed across the RBD and extended notably into non-RBM regions, showing a pattern similar to the dispersed escape landscape revealed by DMS. This correspondence suggests that the immune escape potential identified in vitro may be associated with the distribution of some naturally circulating mutations, and the escape landscape obtained from DMS can partly reflect adaptive selection pressures in the real world. Nevertheless, partial mismatches exist between in vitro escape potency and natural circulation frequency: sites with robust in vitro escape effects such as 391 and 532 rarely appeared in surveillance data, while moderate escape residues including 346 and 493 persisted widely in circulating strains. Certain prevalent natural substitutions such as 455 lacked detectable escape capacity in our screening. These differences indicate that DMS escape scores represent in vitro phenotypes obtained under a specific viral genetic background and serum selection pressure, and mainly reflect the relative potential of individual amino acid substitutions to evade antibody neutralization. In contrast, changes in mutation frequencies in nature are population evolutionary outcomes shaped jointly by immune selection, viral functional constraints, transmission fitness, and epidemiological processes. Therefore, the overlap between DMS-identified escape mutations and circulating mutations only suggests that immune escape may favor the selection and retention of these mutations during natural transmission but does not prove that their increased frequencies are directly caused by immune escape. This discrepancy indicates real-world viral evolution is governed by multiple constraints beyond immune pressure, including ACE2 binding affinity, spike structural stability, replicative fitness and upper respiratory tract transmissibility [34,35,36,37]. Therefore, the association between DMS escape signals and natural mutation frequencies observed in this study should be regarded as indirect evidence supporting the involvement of immune selection in viral evolution, and DMS results alone cannot be used to predict whether a given variant will become dominant. Future studies should further integrate viral entry and replication fitness assays, time-series surveillance, phylogenetic analyses, independent recurrent emergence across different regions, and transmission dynamic studies to evaluate the actual contribution of immune escape to changes in mutation frequency.

Comparisons with the ancestral Wuhan-Hu-1 strain confirmed substitutions reverting to ancestral amino acids at key escape residues can also confer neutralization evasion. This observation demonstrates antigenic evolution does not follow a unidirectional trajectory away from the prototype virus and further highlights that mutational phenotypes are entirely context-dependent on overall spike genetic and structural backgrounds [2,38].

Collectively, our observations support a constrained plasticity model of SARS-CoV-2 antigenic evolution. While extensive RBD residues possess escape potential and tolerate multiple immune-evasive substitutions to supply abundant evolutionary pathways, structural and functional limitations restrict permissible mutations at individual sites, and identical core escape positions are repeatedly selected across distinct variants. Viral antigenic adaptation thus proceeds as targeted optimization within a feasible functional space balanced by infectivity, protein stability and immune selection, rather than random mutagenesis [2,33,34]. Consistent with prior polyclonal serum DMS research, the theoretical landscape of antibody escape remains broad, mutations that combine immune escape potential with the ability to maintain spike function may be more likely to be retained and transmitted during natural circulation [27,28,29]. This study extends this evolutionary framework to the JN.1 and XEC genetic backgrounds and clarifies its manifestations in late-stage Omicron circulation by integrating breakthrough infection serum screening and local long-term genomic surveillance data.

It should be noted that the raw *pv*(*c*) values in this study represent the enrichment level of mutants relative to control conditions before and after antibody selection, and thus are not inherently bounded between 0 and 1. To facilitate visualization of escape magnitudes and relative comparisons across distinct mutations, all raw pv(c) values were converted into normalized relative escape scores ranging from 0 to 1. Accordingly, this 0 to 1 range arises from mathematical normalization; it does not reflect the actual probability that a mutation mediates escape, nor can it be directly converted into a specific fold change in IC50. As this metric relies on shifts in the relative frequencies of mutations within the viral library, its outputs primarily characterize the relative enrichment of each mutation under defined selection pressure. It should therefore be used to compare relative escape effects among mutations and identify potential escape hotspots, rather than interpreted as a direct quantification of absolute neutralization escape capacity. Nevertheless, VSV-G control correction partially mitigates non-specific frequency fluctuations, and standard pseudovirus neutralization assays of representative single mutations further validate the direction of effects identified via DMS.

This study still has several limitations. First, it only analyzed single-amino-acid substitutions in the RBD, without incorporating mutations in the NTD and S2 regions or epistatic interactions among multiple sites. Deletions, insertions, and glycosylation changes in the NTD, as well as mutations in the S2 subunit that affect spike conformational stability and membrane fusion function, may all alter viral antigenic phenotypes and antibody sensitivity. In addition, naturally circulating variants usually carry multiple mutations simultaneously, and their overall escape phenotypes may be jointly determined by synergistic, antagonistic, or compensatory epistatic interactions among mutations. Therefore, the escape maps obtained in this study mainly reflect the direct effects of single RBD mutations in specific variant genetic backgrounds. Some mutations that show weak escape effects or functional impairment when present alone may still acquire functional compensation and produce stronger overall escape when combined with other mutations. Second, the sera used in this study were derived from a specific vaccination and breakthrough-infection background, and the serum sample size was limited. Therefore, the escape maps represent only this specific immune background and cannot comprehensively capture the immune diversity shaped by different ages, vaccine types, and infection intervals. Third, pseudovirus neutralization assays in vitro mainly evaluate the ability of antibodies to block spike-mediated cell entry and identify potential escape mutations that may reduce recognition by existing humoral immunity. Therefore, these results provide evidence, at the level of antibody-mediated inhibition of viral entry, for assessing the in vivo immune escape potential and relative risk of mutations. However, this system cannot fully recapitulate the complex immune protection process in humans. It is difficult to reflect secretory antibody concentrations in the respiratory mucosa, tissue-specific immune responses, and local microenvironments, and it cannot fully evaluate Fc-mediated effector functions such as antibody-dependent cellular cytotoxicity, antibody-dependent phagocytosis, and complement activation. In addition, in vivo protection is also influenced by T-cell immunity, viral tissue tropism, and host baseline conditions. Therefore, the reduced neutralization or enhanced escape observed in this study mainly represents a relative phenotype in the in vitro viral entry step. It can be used for mutation risk screening and relative comparison among mutations, but should not be directly equated with complete loss of in vivo protection, increased disease severity, or true transmission advantage. Fourth, insufficient sequencing read length, experimental replicate variability, and functional defects in spike-mediated entry precluded the quantification of numerous mutations, resulting in non-random missing data in the heatmaps. Mutations with severely impaired function may have been depleted before antibody selection because of reduced pseudovirus packaging or cell-entry efficiency, whereas low initial library abundance, bottleneck effects during infection, and frequency fluctuations between replicates may also cause some true escape mutations to fall below the detection threshold, thereby leading to underestimation or local bias in the escape landscape. Although VSV-G control correction can partly reduce frequency changes unrelated to antibody selection, it cannot eliminate these biases. Therefore, this study applied quality control based on initial mutation abundance, sequencing counts, consistency between experimental replicates, and viral entry function during analysis. Mutations that could not be reliably quantified were excluded from escape score analysis and marked as missing values in the heatmaps, rather than being interpreted as having no escape effect. Thus, regions with concentrated missing data and conclusions based on only a few quantifiable mutations should be interpreted with caution. Fifth, site-level cumulative escape scores are susceptible to distortion by a small number of high-effect mutations, and atypical escape sites require independent experimental validation. Sixth, the association between DMS escape scores and the frequencies of naturally circulating mutations does not indicate a direct causal relationship. Although the overlap between DMS-identified escape mutations and some circulating mutations supports the possibility that immune selection may have contributed to the enrichment of these mutations, this study cannot distinguish the relative contributions of immune escape, overall viral fitness, genetic drift, founder effects, genetic hitchhiking, and epidemiological dynamics to changes in mutation frequency. Therefore, these results cannot be used to determine whether immune escape is the direct, primary, or sole driver of the circulation of these mutations in human populations. The relevant evolutionary mechanisms still require further validation through integrated analyses of viral fitness assays, longitudinal genomic surveillance, phylogenetic analysis, and transmission dynamics. Seventh, ancestral amino acid reversion mutations are currently supported only by phenotypic and sequence evidence, and direct structural biological validation is still lacking. Therefore, this study cannot yet determine whether these reversion mutations affect antibody recognition and neutralization sensitivity by directly altering antibody contact residues, or by modulating local RBD conformation, epitope accessibility, or the conformational equilibrium of the spike trimer. It should be emphasized that restoration of an ancestral amino acid does not mean that the antigenic phenotype has reverted to that of Wuhan-Hu-1. Its functional consequences may depend on the overall genetic backgrounds of JN.1 or XEC and epistatic interactions with other mutations. Therefore, future studies should integrate cryo-electron microscopy, X-ray crystallography, molecular dynamics simulations, and antibody–RBD binding kinetic analyses to dissect the effects of these reversion mutations on antibody binding and spike conformation, thereby validating their molecular mechanisms and evolutionary significance.

In summary, this study systematically mapped the serum escape landscapes of spike RBD mutations in the JN.1 and XEC variants under the specific hybrid immune background of three-dose inactivated vaccination followed by BA.4/5 breakthrough infection, and analyzed the association between in vitro escape phenotypes and naturally circulating mutations. These findings provide experimental evidence for emerging variant risk assessment, vaccine antigen updates, and optimization of immune strategies. Given that continued spike evolution may weaken the effectiveness of existing immune interventions, the post-pandemic era also requires the development of affordable, scalable antiviral drugs targeting conserved viral proteins, as a complement to variant surveillance and vaccine updates and in support of the UN Sustainable Development Goals (UN-SDGs). A recent study reported that 7-hydroxystilbene–coumarin hybrid scaffold compounds can inhibit the SARS-CoV-2 main protease 3CLpro in vitro, with compound 7c showing relatively strong enzymatic inhibitory activity, thereby providing a candidate chemical scaffold for drug development targeting conserved viral replication proteins [39]. Although its safety and antiviral efficacy still require further validation, such drugs may serve as a potential complementary strategy alongside variant surveillance, vaccine updates, and protection of high-risk populations, together contributing to a more sustainable COVID-19 control system.

## Figures and Tables

**Table 1:** Demographic and baseline characteristics of the total participants.

	C001 (N = 4)	C002 (N = 4)	C003 N = 4)	C004 (N = 4)	C005 (N = 4)	C006 (N = 4)	C007 (N = 4)	C008 (N = 4)	C009 (N = 4)	C010 (N = 4)	*p* Value
Mean age (SD), years	52.8 (19.1)	47.5 (18.4)	61.5 (21.7)	57.0 (16.4)	47.8 (10.4)	44.0 (18.9)	42.0 (10.2)	57.3 (13.6)	55.5 (13.2)	60.8 (10.8)	0.6101
Age, years											
18–44	2 (50.0)	2 (50.0)	1 (25.0)	1 (25.0)	2 (50.0)	2 (50.0)	1 (25.0)	1 (25.0)	1 (25.0)	0 (0.0)	0.8619
≥45	2 (50.0)	2 (50.0)	3 (75.0)	3 (75.0)	2 (50.0)	2 (50.0)	3 (75.0)	3 (75.0)	3 (75.0)	4 (100.0)	
Sex											
Male	3 (75.0)	3 (75.0)	1 (25.0)	1 (25.0)	1 (25.0)	1 (25.0)	4 (100.0)	1 (25.0)	2 (50.0)	3 (75.0)	0.2133
Female	1 (25.0)	1 (25.0)	3 (75.0)	3 (75.0)	3 (75.0)	3 (75.0)	0 (0.0)	3 (75.0)	2 (50.0)	1 (25.0)	
Time from the last dose of vaccination to breakthrough infection											
Median (IQR), months	15.0 (12.5–17.1)	18.5 (17.2–18.7)	11.6 (9.9–14.5)	13.5 (13.1–14.3)	14.3 (11.9–16.9)	16.0 (13.0–18.5)	18.5 (16.7–18.7)	13.4 (13.0–14.2)	14.3 (11.5–17.1)	14.9 (13.6–16.7)	0.4519
≤12 months	1 (25.0)	0 (0.0)	0 (0.0)	0 (0.0)	1 (25.0)	1 (25.0)	1 (25.0)	1 (25.0)	1 (25.0)	0 (0.0)	0.7768
>12 months	3 (75.0)	4 (100.0)	4 (100.0)	4 (100.0)	3 (75.0)	3 (75.0)	3 (75.0)	3 (75.0)	3 (75.0)	4 (100.0)	
Time from the blood sample collection to breakthrough infection											
Median (IQR), months	22.0 (19.7–24.0)	25.6 (24.4–25.8)	18.9 (17.1–21.9)	20.6 (20.3–21.3)	21.4 (18.8–24.0)	23.1 (20.1–25.5)	25.6 (23.9–25.8)	20.6 (20.2–21.3)	21.2 (18.5–24.1)	22.0 (20.6–23.9)	0.6169

Each group included four participants (N). Data are mean (SD), n (%) or median (IQR). The two-sided x’ test or Fisher’s exact test was used for categorical data. The two-sided Kruskal–Wallis test was used for continuous data.

## Data Availability

All sequences are publicly available at the National Center for Biotechnology Information under project PRJNA1415785. The relevant code is available at https://github.com/CWShao729 (accessed on 27 June 2026).

## Supplementary Materials

The following supporting information can be downloaded at https://www.mdpi.com/article/10.3390/microorganisms14091872/s1, Figure S1: Flowchart for constructing a genotype-phenotype associated spike protein DMS library; Figure S2: Composition of the Omicron JN.1 and XEC RBD mutational library; Figure S3: Correlation between JN.1 and XEC RBD mutations mediating serum escape effects; Figure S4: Validation pseudovirus neutralization assays for JN.1 and XEC spike mutants that escape serum neutralization; Figure S5: Proportion of serum escape sites in the RBD of JN.1 and XEC; Figure S6: Mutational complexity and RBM enrichment of serum escape sites in the RBD of JN.1 and XEC; Figure S7: Distribution of JN.1 and XEC RBD serum escape mutations and mutations observed in epidemic variants in 2024 and 2025.

## References

[1] Reynolds C.J., Pade C., Gibbons J.M., Otter A.D., Lin K.M., Muñoz Sandoval D., Pieper F.P., Butler D.K., Liu S., Joy G. (2022). Immune boosting by B.1.1.529 (Omicron) depends on previous SARS-CoV-2 exposure. Science.

[2] Cao Y., Jian F., Wang J., Yu Y., Song W., Yisimayi A., Wang J., An R., Chen X., Zhang N. (2023). Imprinted SARS-CoV-2 humoral immunity induces convergent Omicron RBD evolution. Nature.

[3] Harvey W.T., Carabelli A.M., Jackson B., Gupta R.K., Thomson E.C., Harrison E.M., Ludden C., Reeve R., Rambaut A., Peacock S.J. (2021). SARS-CoV-2 variants, spike mutations and immune escape. Nat. Rev. Microbiol..

[4] Cao Y., Wang J., Jian F., Xiao T., Song W., Yisimayi A., Huang W., Li Q., Wang P., An R. (2022). Omicron escapes the majority of existing SARS-CoV-2 neutralizing antibodies. Nature.

[5] Liu L., Iketani S., Guo Y., Chan J.F., Wang M., Liu L., Luo Y., Chu H., Huang Y., Nair M.S. (2022). Striking antibody evasion manifested by the Omicron variant of SARS-CoV-2. Nature.

[6] Qu P., Evans J.P., Faraone J.N., Zheng Y.M., Carlin C., Anghelina M., Stevens P., Fernandez S., Jones D., Lozanski G. (2023). Enhanced neutralization resistance of SARS-CoV-2 Omicron subvariants BQ.1, BQ.1.1, BA.4.6, BF.7, and BA.2.75.2. Cell Host Microbe.

[7] Dadonaite B., Brown J., McMahon T.E., Farrell A.G., Figgins M.D., Asarnow D., Stewart C., Lee J., Logue J., Bedford T. (2024). Spike deep mutational scanning helps predict success of SARS-CoV-2 clades. Nature.

[8] Starr T.N., Greaney A.J., Addetia A., Hannon W.W., Choudhary M.C., Dingens A.S., Li J.Z., Bloom J.D. (2021). Prospective mapping of viral mutations that escape antibodies used to treat COVID-19. Science.

[9] Crawford K.H.D., Eguia R., Dingens A.S., Loes A.N., Malone K.D., Wolf C.R., Chu H.Y., Tortorici M.A., Veesler D., Murphy M. (2020). Protocol and Reagents for Pseudotyping Lentiviral Particles with SARS-CoV-2 Spike Protein for Neutralization Assays. Viruses.

[10] VanBlargan L.A., Errico J.M., Halfmann P.J., Zost S.J., Crowe J.E., Purcell L.A., Kawaoka Y., Corti D., Fremont D.H., Diamond M.S. (2022). An infectious SARS-CoV-2 B.1.1.529 Omicron virus escapes neutralization by therapeutic monoclonal antibodies. Nat. Med..

[11] Planas D., Saunders N., Maes P., Guivel-Benhassine F., Planchais C., Buchrieser J., Bolland W.H., Porrot F., Staropoli I., Lemoine F. (2022). Considerable escape of SARS-CoV-2 Omicron to antibody neutralization. Nature.

[12] Fowler D.M., Fields S. (2014). Deep mutational scanning: A new style of protein science. Nat. Methods.

[13] Taft J.M., Weber C.R., Gao B., Ehling R.A., Han J., Frei L., Metcalfe S.W., Overath M.D., Yermanos A., Kelton W. (2022). Deep mutational learning predicts ACE2 binding and antibody escape to combinatorial mutations in the SARS-CoV-2 receptor-binding domain. Cell.

[14] Wu L., Zhou L., Mo M., Liu T., Wu C., Gong C., Lu K., Gong L., Zhu W., Xu Z. (2022). SARS-CoV-2 Omicron RBD shows weaker binding affinity than the currently dominant Delta variant to human ACE2. Signal Transduct. Target. Ther..

[15] Jian F., Wec A.Z., Feng L., Yu Y., Wang L., Wang P., Yu L., Wang J., Hou J., Berrueta D.M. (2025). Viral evolution prediction identifies broadly neutralizing antibodies to existing and prospective SARS-CoV-2 variants. Nat. Microbiol..

[16] Robbiani D.F., Gaebler C., Muecksch F., Lorenzi J.C.C., Wang Z., Cho A., Agudelo M., Barnes C.O., Gazumyan A., Finkin S. (2020). Convergent antibody responses to SARS-CoV-2 in convalescent individuals. Nature.

[17] Goel R.R., Painter M.M., Apostolidis S.A., Mathew D., Meng W., Rosenfeld A.M., Lundgreen K.A., Reynaldi A., Khoury D.S., Pattekar A. (2021). mRNA vaccines induce durable immune memory to SARS-CoV-2 and variants of concern. Science.

[18] Yisimayi A., Song W., Wang J., Jian F., Yu Y., Chen X., Xu Y., Yang S., Niu X., Xiao T. (2024). Repeated Omicron exposures override ancestral SARS-CoV-2 immune imprinting. Nature.

[19] Dadonaite B., Crawford K.H.D., Radford C.E., Farrell A.G., Yu T.C., Hannon W.W., Zhou P., Andrabi R., Burton D.R., Liu L. (2023). A pseudovirus system enables deep mutational scanning of the full SARS-CoV-2 spike. Cell.

[20] Shao C., Yang L., Xiao C., Huang Y., Wang X., Chen L., Liu P., Chen S., Wei M., Jia S. (2026). Deep mutational scanning reveals the antibody escape and infectivity landscape of SARS-CoV-2 Omicron JN.1 and XEC receptor-binding domains. Emerg. Microbes Infect..

[21] Jung Y., Han D. (2022). BWA-MEME: BWA-MEM emulated with a machine learning approach. Bioinformatics.

[22] Li H., Handsaker B., Wysoker A., Fennell T., Ruan J., Homer N., Marth G., Abecasis G., Durbin R. (2009). The Sequence Alignment/Map format and SAMtools. Bioinformatics.

[23] Thorvaldsdóttir H., Robinson J.T., Mesirov J.P. (2013). Integrative Genomics Viewer (IGV): High-performance genomics data visualization and exploration. Brief. Bioinform..

[24] Bushnell B., Rood J., Singer E. (2017). BBMerge—Accurate paired shotgun read merging via overlap. PLoS ONE.

[25] Kechin A., Boyarskikh U., Kel A., Filipenko M. (2017). cutPrimers: A New Tool for Accurate Cutting of Primers from Reads of Targeted Next Generation Sequencing. J. Comput. Biol..

[26] Zorita E., Cuscó P., Filion G.J. (2015). Starcode: Sequence clustering based on all-pairs search. Bioinformatics.

[27] Greaney A.J., Loes A.N., Crawford K.H.D., Starr T.N., Malone K.D., Chu H.Y., Bloom J.D. (2021). Comprehensive mapping of mutations in the SARS-CoV-2 receptor-binding domain that affect recognition by polyclonal human plasma antibodies. Cell Host Microbe.

[28] Greaney A.J., Starr T.N., Barnes C.O., Weisblum Y., Schmidt F., Caskey M., Gaebler C., Cho A., Agudelo M., Finkin S. (2021). Mapping mutations to the SARS-CoV-2 RBD that escape binding by different classes of antibodies. Nat. Commun..

[29] Dadonaite B., Harari S., Larsen B.B., Kampman L., Harteloo A., Elias-Warren A., Chu H.Y., Bloom J.D. (2025). Spike mutations that affect the function and antigenicity of recent KP.3.1.1-like SARS-CoV-2 variants. J. Virol..

[30] Ramasamy R. (2025). Perspective Overview of Changing Population Immunity to COVID-19 in the Context of Infection, Vaccination, and Emerging SARS-CoV-2 Variants. Pathogens.

[31] Greaney A.J., Starr T.N., Gilchuk P., Zost S.J., Binshtein E., Loes A.N., Hilton S.K., Huddleston J., Eguia R., Crawford K.H.D. (2021). Complete Mapping of Mutations to the SARS-CoV-2 Spike Receptor-Binding Domain that Escape Antibody Recognition. Cell Host Microbe.

[32] Watanabe Y., Allen J.D., Wrapp D., McLellan J.S., Crispin M. (2020). Site-specific glycan analysis of the SARS-CoV-2 spike. Science.

[33] Herzig J.C., Magwira M.L., Lovell S.C. (2026). Structural Constraints Acting on the SARS-CoV-2 Spike Protein Reveal Limited Space for Viral Adaptation. Genome Biol. Evol..

[34] Feng Z., Sang Z., Xiang Y., Escalera A., Weshler A., Schneidman-Duhovny D., García-Sastre A., Shi Y. (2026). One thousand SARS-CoV-2 antibody structures reveal convergent binding and near-universal immune escape. Cell Syst..

[35] Bloom J.D., Neher R.A. (2023). Fitness effects of mutations to SARS-CoV-2 proteins. Virus Evol..

[36] Ma W., Fu H., Jian F., Cao Y., Li M. (2023). Immune evasion and ACE2 binding affinity contribute to SARS-CoV-2 evolution. Nat. Ecol. Evol..

[37] Peacock T.P., Goldhill D.H., Zhou J., Baillon L., Frise R., Swann O.C., Kugathasan R., Penn R., Brown J.C., Sanchez-David R.Y. (2021). The furin cleavage site in the SARS-CoV-2 spike protein is required for transmission in ferrets. Nat. Microbiol..

[38] Starr T.N., Greaney A.J., Hannon W.W., Loes A.N., Hauser K., Dillen J.R., Ferri E., Farrell A.G., Dadonaite B., McCallum M. (2022). Shifting mutational constraints in the SARS-CoV-2 receptor-binding domain during viral evolution. Science.

[39] Khamto N., Pruksaphon K., Akkravijitkul N., Choomongkol V., Patnin S., Meepowpan P. (2026). Design, Synthesis and Computational Insights of 7-Hydroxystilbene-Coumarin Hybrid Scaffolds as SARS-CoV-2 3CLpro Inhibitors. J. Mol. Struct..

