# Supplementary material for: Serum Escape Landscape of SARS-CoV-2 Omicron JN.1 and XEC RBD Under COVID-19 Vaccine Breakthrough Immunity in China
Source: Microorganisms. 2026 Aug 23;14(9):1872. doi: 10.3390/microorganisms14091872 (PMC13609519; doi:10.3390/microorganisms14091872)
Supplement: Supplementary file 1 [file microorganisms-14-01872-s001.zip › Supplementary_Figures.pdf]

Supplementary Figures

Paper title

Serum escape landscape of SARS-CoV-2 Omicron JN.1 and XEC RBD under COVID-19 vaccine breakthrough immunity in China

Table of Contents

Figure S1. Flowchart for constructing a genotype-phenotype associated spike protein DMS library.

Figure S2. Composition of the Omicron JN.1 and XEC RBD mutational library.

Figure S3. Correlation between JN.1 and XEC RBD mutations mediating serum escape effects.

Figure S4. Validation pseudovirus neutralization assays for JN.1 and XEC spike mutants that escape serum neutralization.

Figure S5. Proportion of serum escape sites in the RBD of JN.1 and XEC.

Figure S6. Mutational complexity and RBM enrichment of serum escape sites in the RBD of JN.1 and XEC.

Figure S7. Distribution of JN.1 and XEC RBD serum escape mutations and mutations observed in epidemic variants in 2024 and 2025.

Figure S1. Flowchart for constructing a genotype-phenotype associated spike protein DMS library.

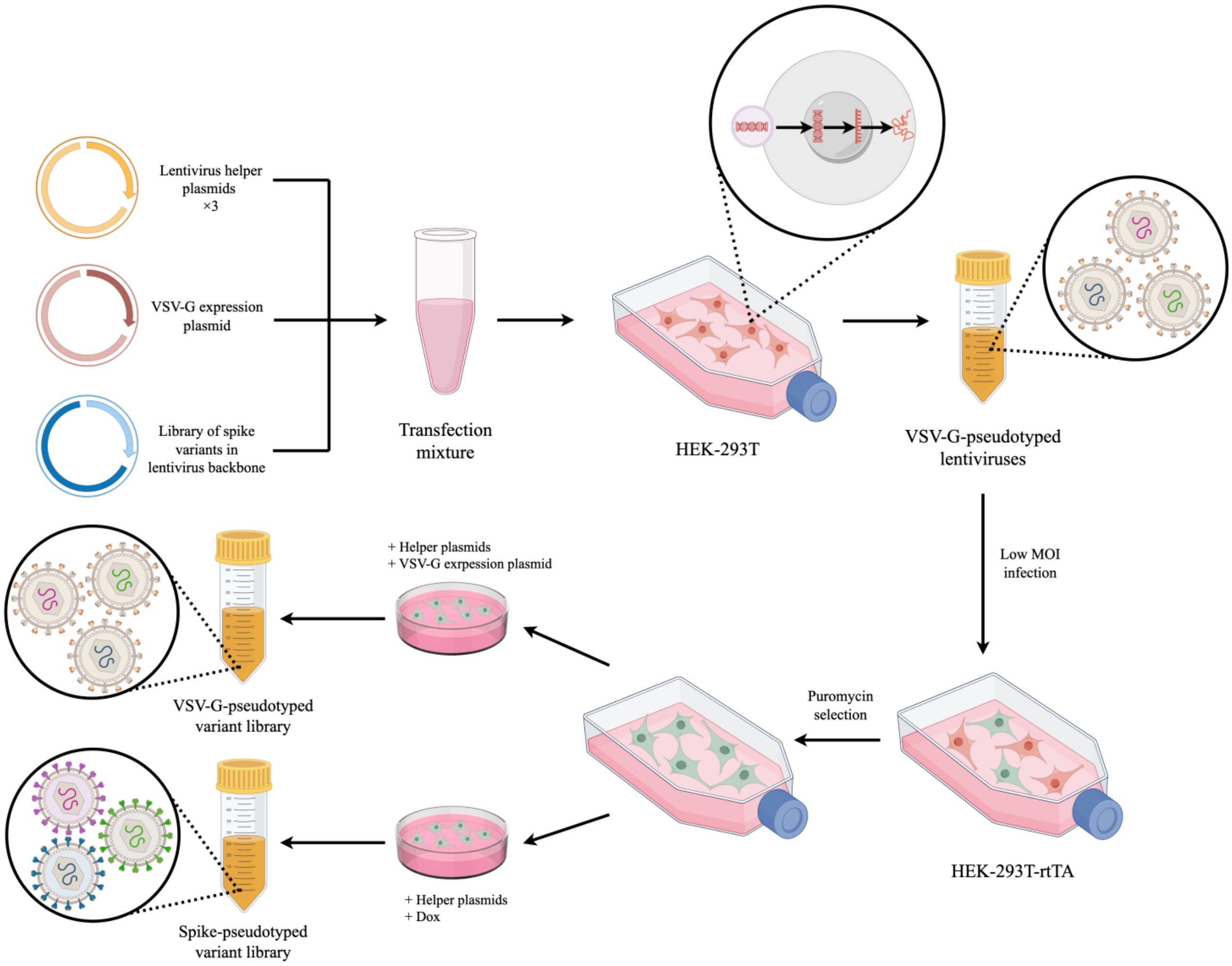

Lentiviral backbone plasmids encoding spike variants were co-transfected into HEK-293T cells together with VSV-G expression and lentiviral helper plasmids to generate VSV-G-pseudotyped lentiviruses carrying spike genes. HEK-293T-rtTA cells were then infected with a low MOI, and a cell population of integrated mutants was obtained by selection with puromycin. Base on this, spike-pseudotyped lentiviral libraries were produced by transfecting helper plasmids in the presence of doxycycline to induce spike expression. In parallel, VSV-G–pseudotyped lentiviral libraries were generated by co-transfecting the VSV-G expression plasmid together with helper plasmids.

Figure S2. Composition of the Omicron JN.1 and XEC RBD mutational library.

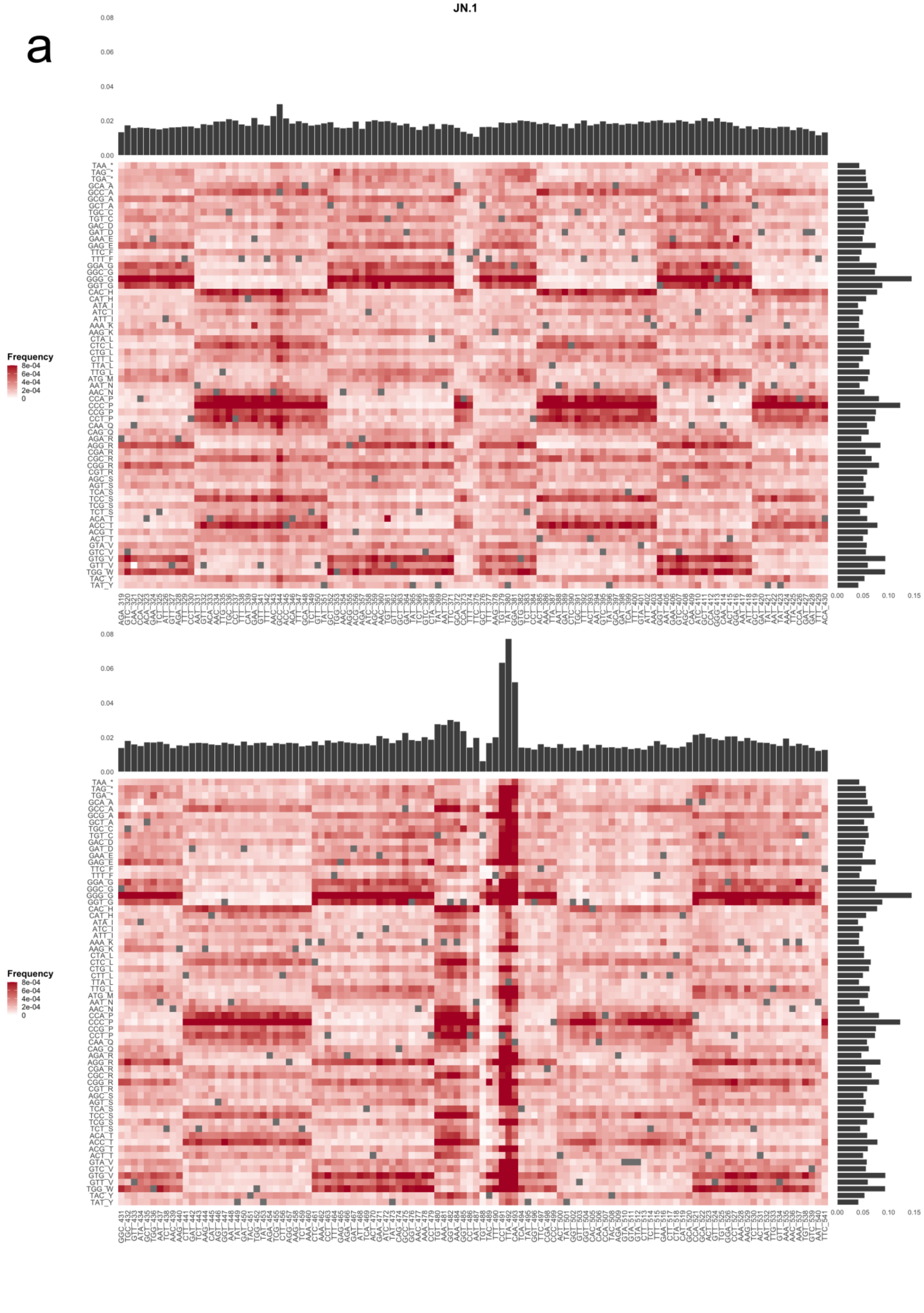

b

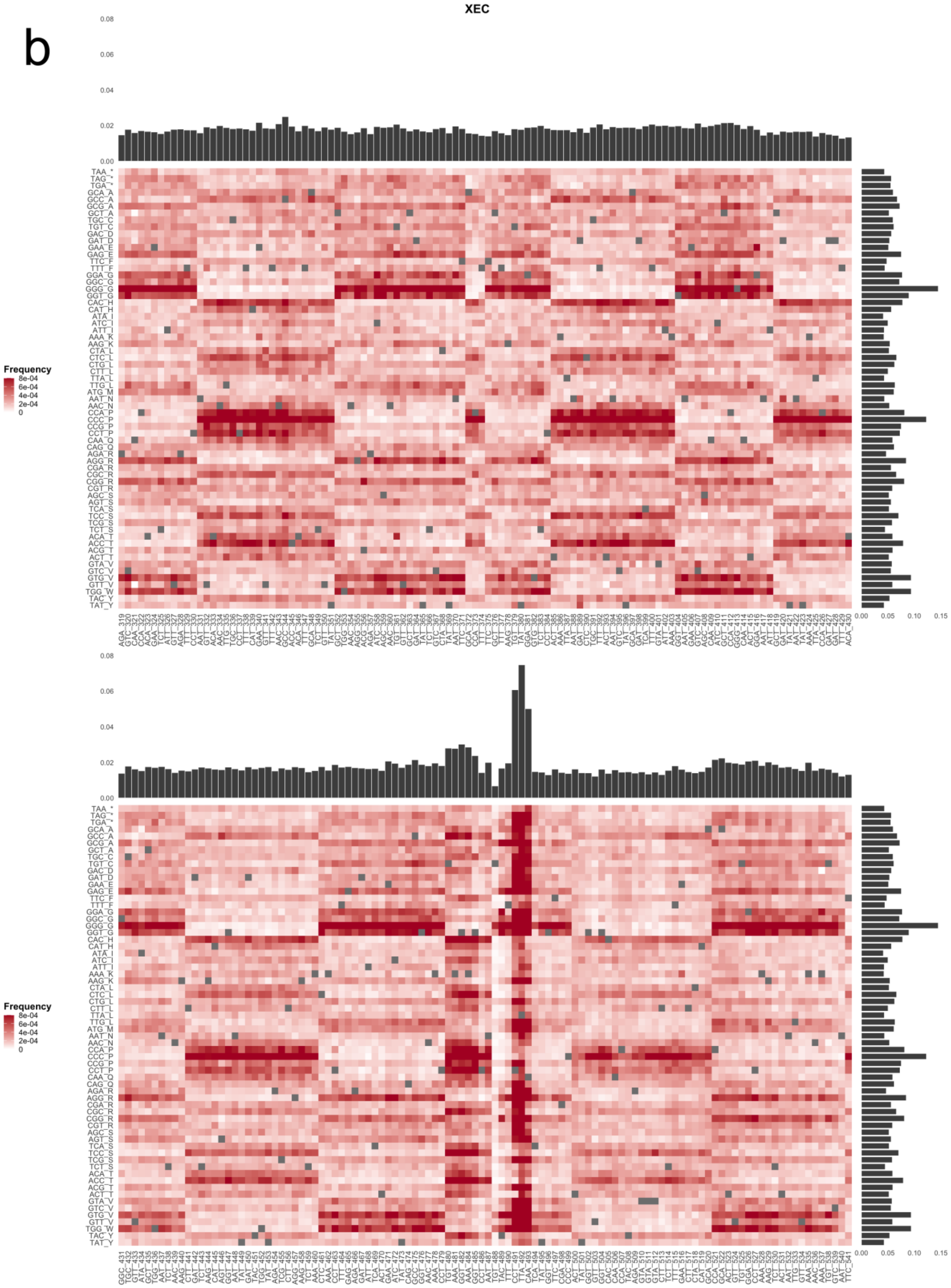

The heatmaps show the frequency distribution of designed codon substitutions in the RBD (amino acid residues 319-541) of the JN.1 (a) and XEC (b) spike proteins in the library. The partition is based on

residue 430 (top: 319-430; bottom: 431-541). Each column represents an RBD site, and each row represents a specific codon mutation designed at that site. Colors indicate the observed frequency of the corresponding mutation in sequencing. The bars above each heatmap represent the sum of the frequencies of all substitution mutations at each site, and the bars on the right represent the cumulative frequency of each codon mutation across all sites. Wild-type counts have been excluded from the statistics. Gray squares indicate wild-type codons at each site.

Figure S3. Correlation between JN.1 and XEC RBD mutations mediating serum escape effects.

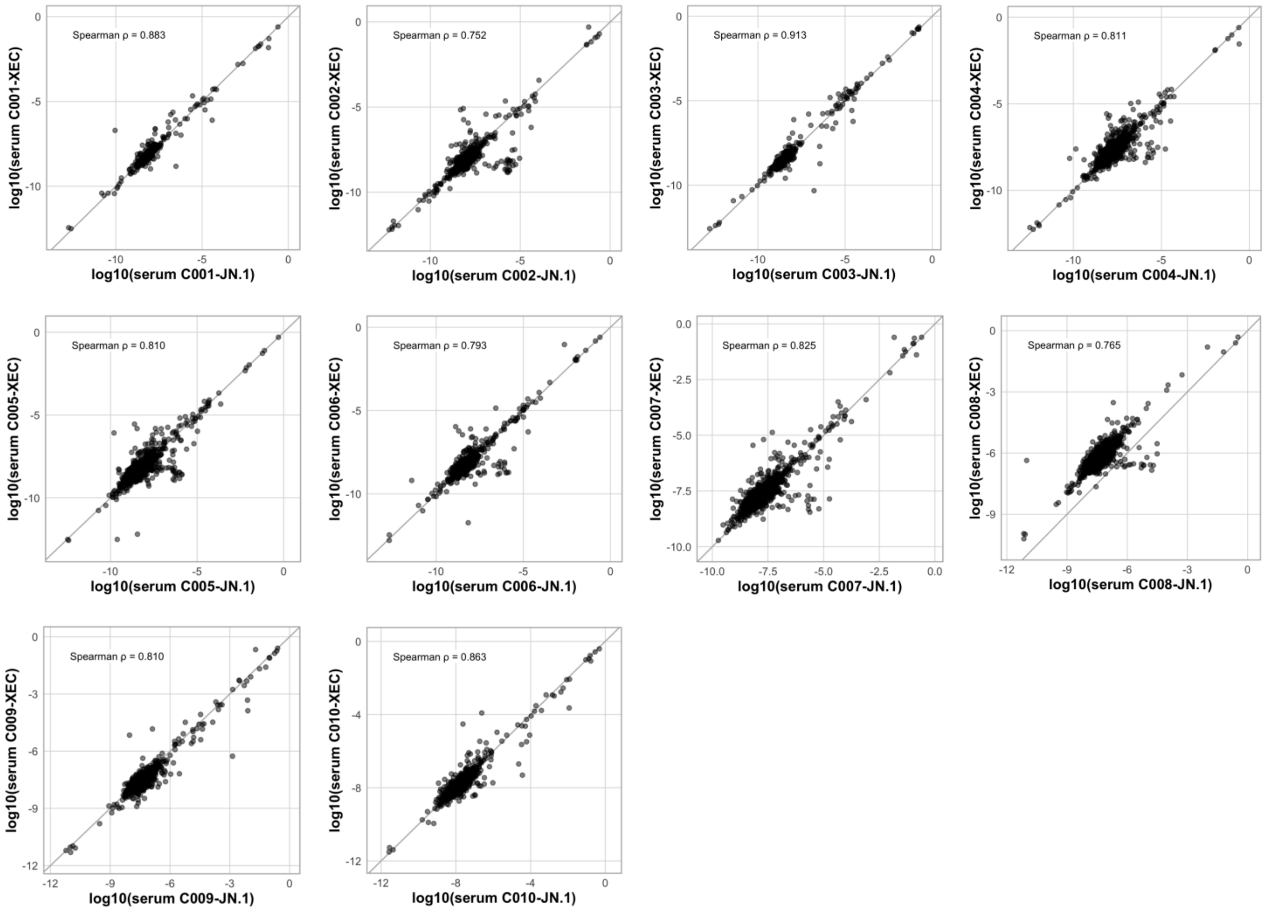

Each panel shows the correlation between JN.1 and XEC spike RBD mutation-mediated serum escape effect in single serum conditions.

Figure S4. Validation pseudovirus neutralization assays for JN.1 and XEC spike mutants that escape serum neutralization.

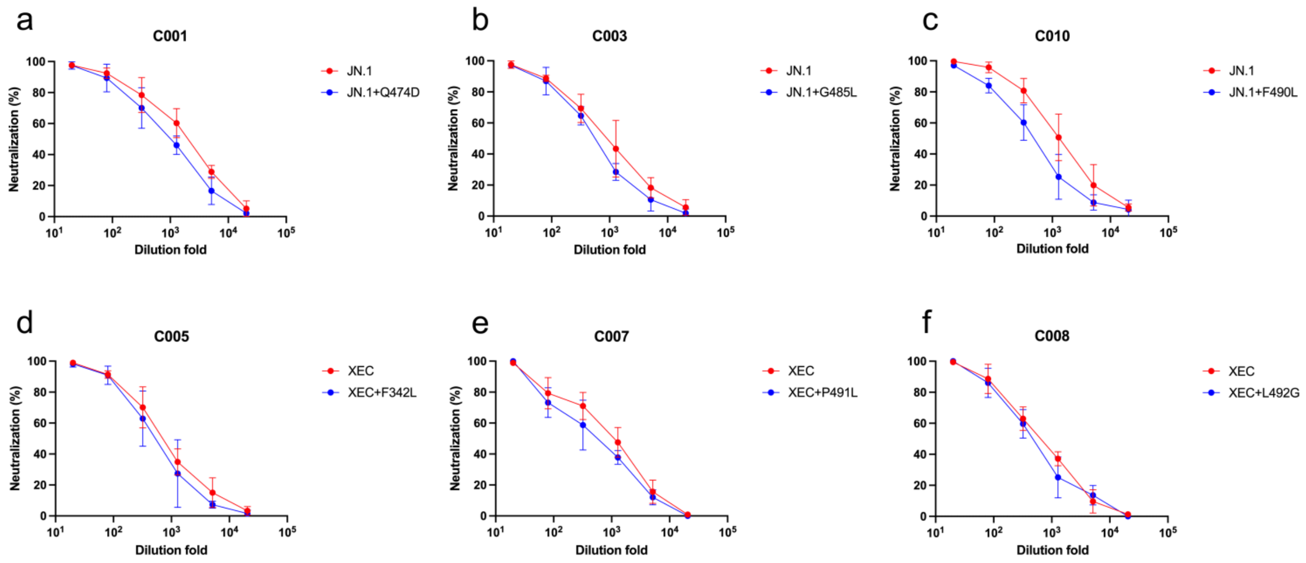

Pseudovirus neutralization assay was conducted to verify the JN.1 (a-c) and XEC (d-f) mutants, which were predicted to evade neutralization by different sera based on DMS results. The error bar represents the standard error of two technical replications.

Figure S5. Proportion of serum escape sites in the RBD of JN.1 and XEC.

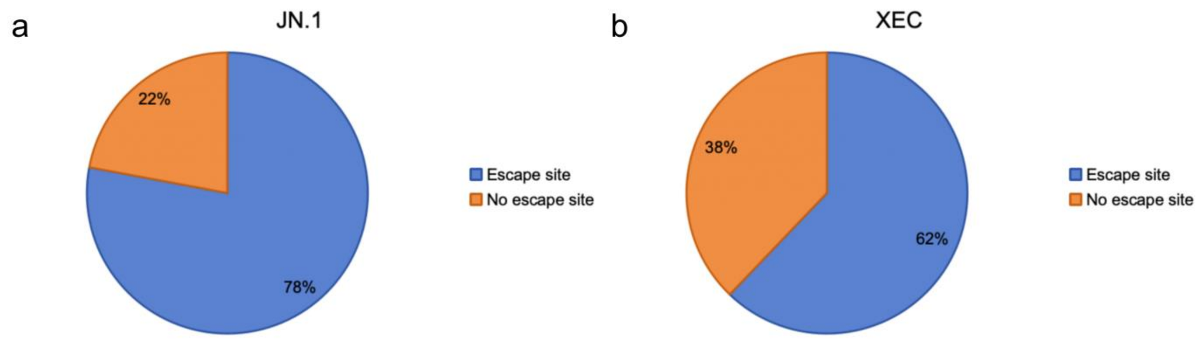

The fraction of JN.1 (a) and XEC (b) RBD residues classified as serum escape sites or non-escape sites based on DMS analyses. Escape amino-acid mutations were defined as substitutions exhibiting measurable serum escape effects in at least 5 out of the 10 tested sera. Escape sites were subsequently defined as residues harboring five or more distinct escape amino-acid substitutions.

Figure S6. Mutational complexity and RBM enrichment of serum escape sites in the RBD of JN.1 and XEC.

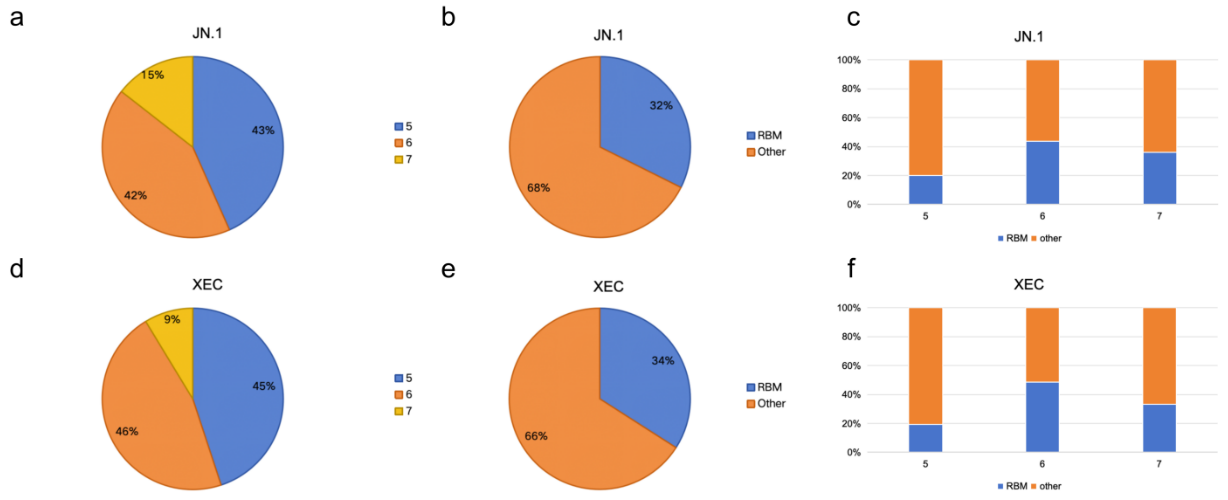

Escape sites were defined as RBD residues harboring at least five distinct amino-acid substitutions that exhibited serum escape in deep mutational scanning assays. Pie charts showing the distribution of escape sites grouped by mutational complexity (five, six, or seven distinct amino-acid substitutions per site) in JN.1 (a) and XEC (d). Pie charts showing the overall proportion of receptor-binding motif (RBM; residues 483–506) and non-RBM sites among all escape sites in JN.1 (b) and XEC (e). Stacked bar plots showing the fraction of RBM and non-RBM sites within each mutational complexity group (5, 6, or 7 substitutions) for JN.1 (c) and XEC (f).

Figure S7. Distribution of JN.1 and XEC RBD serum escape mutations and mutations observed in epidemic variants in 2024 and 2025.

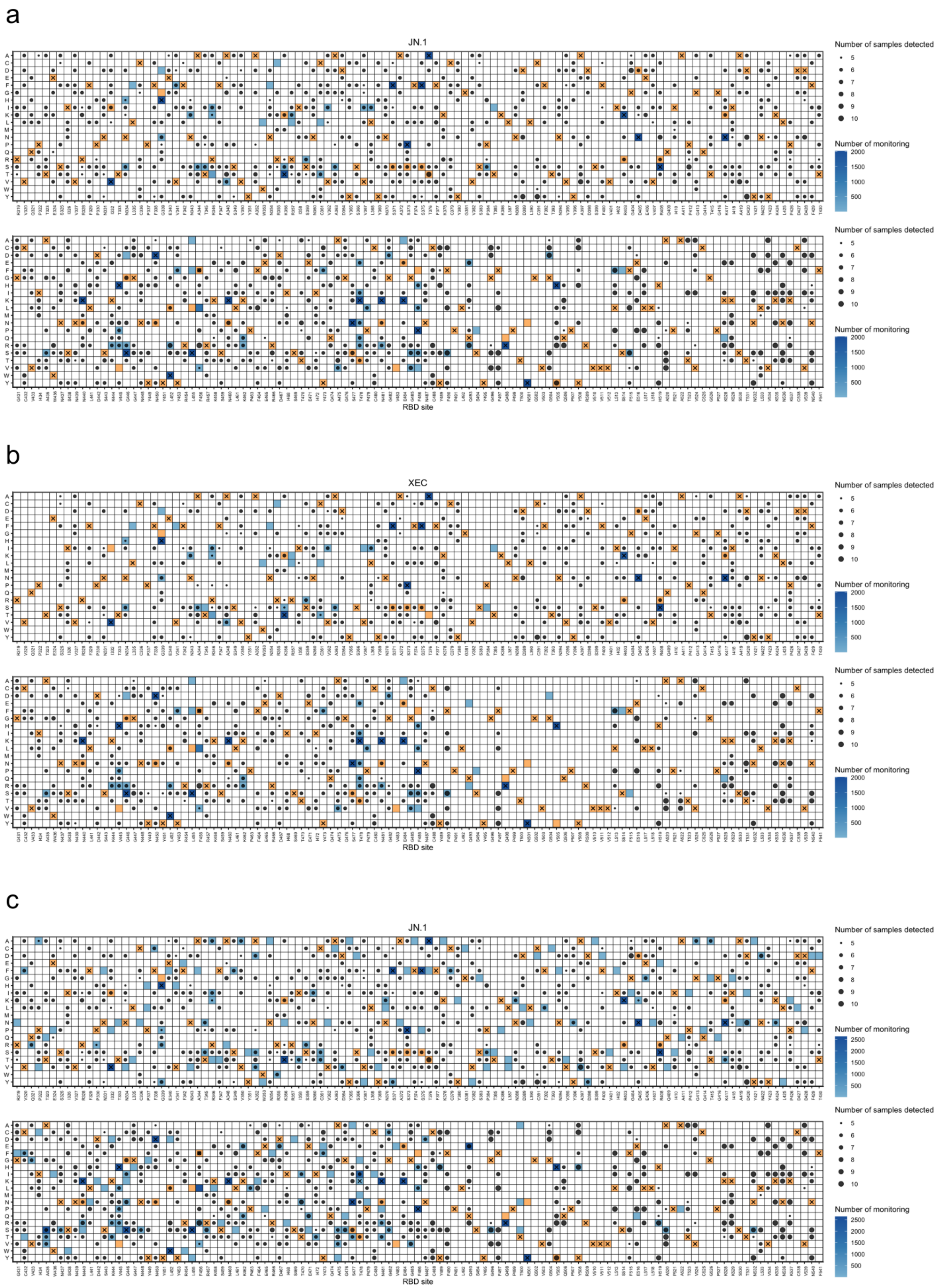

d

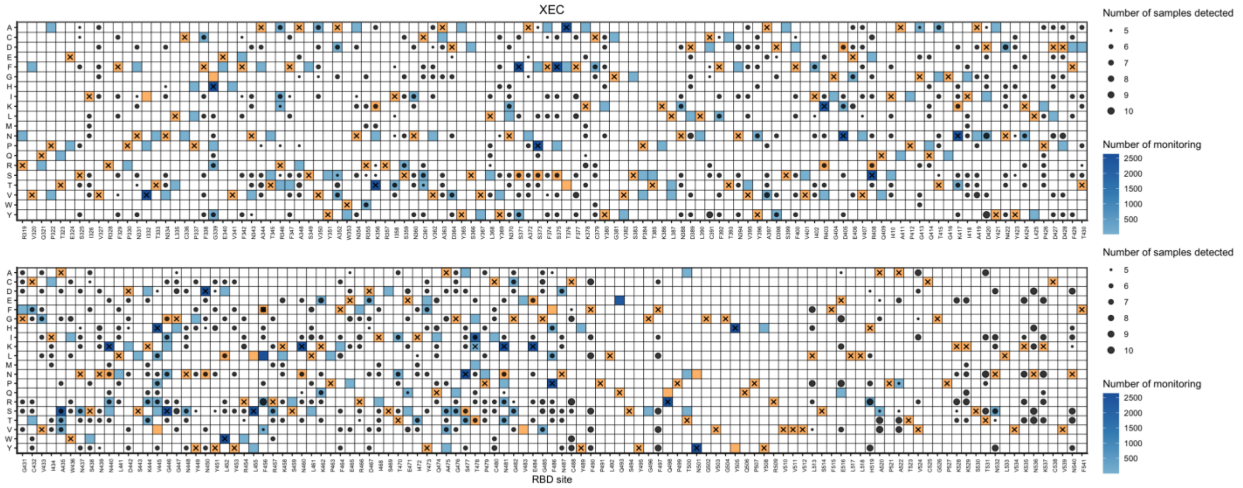

Comprehensive mapping of amino-acid substitutions in the RBD (residues 319-541) of the JN.1 (a, c) and XEC (b, d) that mediate escape from serum neutralizing antibodies, together with mutations observed in epidemic variants detected through genomic surveillance in 2024 (a, b) and 2025 (c, d). The x-axis indicates RBD residue positions, and the y-axis represents amino acid substitutions. Each square corresponds to a single amino acid substitution at a specific RBD site. The intensity of the blue square reflects the frequency of this mutation within infection the surveillance data for epidemic variants in Jiangsu Province during that year (darker shades indicate higher prevalence). The size of the black dot indicates the number of samples out of 10 breakthrough infection serum samples in which the mutation produced a measurable escape effect. Orange squares represent the reference amino acids at each RBD position in the Wuhan-Hu-1 (SARS-CoV-2 ancestral strain), while markers with “×” indicate the amino acids present at each RBD position in JN.1 and XEC.
